# Progression in translational research on spinal cord injury based on microenvironment imbalance

**DOI:** 10.1038/s41413-022-00199-9

**Published:** 2022-04-08

**Authors:** Baoyou Fan, Zhijian Wei, Shiqing Feng

**Affiliations:** 1 Department of Orthopedics, Tianjin Medical University General Hospital, International Science and Technology Cooperation Base of Spinal Cord Injury, Tianjin Key Laboratory of Spine and Spinal Cord Injury, 154 Anshan Road, Heping District, Tianjin, 300052 China; 2 Department of Orthopaedics, Qilu Hospital, Shandong University Centre for Orthopaedics, Cheeloo College of Medicine, Shandong University, Jinan, Shandong 250012 China

**Keywords:** Neurophysiology, Pathogenesis

## Abstract

Spinal cord injury (SCI) leads to loss of motor and sensory function below the injury level and imposes a considerable burden on patients, families, and society. Repair of the injured spinal cord has been recognized as a global medical challenge for many years. Significant progress has been made in research on the pathological mechanism of spinal cord injury. In particular, with the development of gene regulation, cell sequencing, and cell tracing technologies, in-depth explorations of the SCI microenvironment have become more feasible. However, translational studies related to repair of the injured spinal cord have not yielded significant results. This review summarizes the latest research progress on two aspects of SCI pathology: intraneuronal microenvironment imbalance and regenerative microenvironment imbalance. We also review repair strategies for the injured spinal cord based on microenvironment imbalance, including medications, cell transplantation, exosomes, tissue engineering, cell reprogramming, and rehabilitation. The current state of translational research on SCI and future directions are also discussed. The development of a combined, precise, and multitemporal strategy for repairing the injured spinal cord is a potential future direction.

## Introduction

Spinal cord injury (SCI) includes traumatic and nontraumatic SCI. Following traumatic SCI, the most common type of SCI in the clinic, regeneration of the spinal cord is poor, and there are no effective treatments available for SCI. Thus, in this review, we focus on traumatic SCI. Global epidemiological data for SCI are still lacking, but many countries and regions have established SCI databases.^1,2^ According to a recent comprehensive epidemiological study, in 2016, the incidence of SCI was 0.93 million (0.78–1.16 million), and the prevalence was 27.04 million (24.98–30.15 million) worldwide.^3^ The incidence of SCI is higher among males than among females.^4,5^ The age-standardized incidence and prevalence of SCI did not significantly increase between 1990 and 2016, but the absolute number of people with SCI increased. The aging of the global population may have increased the incidence of SCI among aging people.^4^ The most common cause of SCI is falls, which is consistent with to our report from Tianjin, China.^6^ Additionally, patients with SCI die 2 to 5 times earlier than people without SCI, and the mortality rate of SCI depends on the level and severity of the injury.^4^ A study from Canada reported that the net cost of SCI is $336 000 per person and that costs in the first year after SCI are the highest.^7^ However, complications of SCI (atelectasis, pneumonia, venous thromboembolism, depression, etc.) further influence quality of life and increase the economic burden of the disease.^8^ Thus, SCI is a global health problem.

## Animal models

**Fig. 1:**
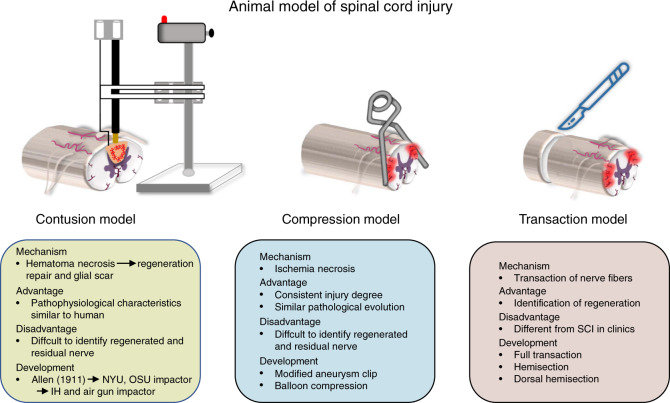
Animal models of SCI. This figure illustrates the mechanisms, advantages, disadvantages, and development of the three SCI animal models

Three models are routinely used to study SCI, i.e., contusion, compression, and transection models (Fig. 1). As each model has specific characteristics, researchers can choose the most appropriate model for their experimental design.

The contusion model is generally established by applying a contusive force generated by an impactor to the spinal cord. In this model, a vertical force instantly impacts the spinal cord. The central artery of the spinal cord and the microvasculature that supplies blood to the spinal cord are immediately destroyed, initiating the acute phase of injury.^9^ Subsequently, local hemorrhage causes an influx of inflammatory cells, vasoactive peptides, and cytokines into the spinal cord. Activation of proapoptotic signaling pathways, changes in cell permeability, and ischemic injury contribute to the loss of a large number of functional neurons and demyelination, which completely disrupt the focal spinal cord microenvironment.^10^ The inflammatory cells released in response to the destruction of blood vessels produce inflammatory cytokines, such as TNF-α and IL-1β, which continue to exert an inflammatory effect in the injured area beyond the subacute phase.^11^ A cascade of reactions triggered by inflammation after injury, combined with destruction of the blood–spinal cord barrier, gradually aggravate spinal cord swelling and further mechanical compression, which also leads to subsequent secondary damage to the spinal cord. In 1911, Allen first established a contusion model by applying a vertical force to the spinal cord. Since this model exhibited similar pathophysiological characteristics and changes as those observed in humans with SCI, it was helpful for exploring the pathological mechanism of SCI. This model was once the preferred and most widely used model in SCI research.^12^ However, it is difficult to achieve a consistent impact site using this model, and the impactor has been continuously improved. The New York University impactor^13^ and Ohio State University impactor^14^ impact the spinal cord with a weight and a solenoid, respectively, with the aid of computers to achieve contusion injury, allowing researchers to select the degree of injury in advance by adjusting the biomechanical parameters. The infinite horizon impactor,^15^ the most widely used contusion impactor, can be used to achieve a reproducible and uniform injury via precise control of strike parameters, such as speed, strength, and impulse, without touching the spinal cord in advance. More recently, a new type of air gun impactor^16^ that damages the white matter of the spinal cord via precise adjustment of the air pressure has emerged. The latest research data has also revealed the high similarity between this type of injury and moderate SCI in patients. Nevertheless, the difficulty in identifying regenerating axons is a limitation of the application of the contusion model in tissue engineering research.^17^

The compression model was first established by Tator and Rivlin, who injured the spinal cord with a modified aneurysm clip to mimic spinal stenosis or traumatic spinal cord compression injury in clinical patients.^18^ The equipment used to generate this model, such as forceps and compression balloons, have since been improved.^19,20^ The degree of injury can be controlled by adjusting the time or the force of compression to explore the time window for surgical decompression.^21^ The characteristics of microenvironment imbalance in this model depend on the duration and extent of the injury. After a compressive force is applied to the spinal cord, local ischemic injury occurs, and then fragile neurons undergo degeneration and necrosis within a short period after ischemia.^22^ As compression is generally relieved, devastating spinal cord ischemia–reperfusion injury (SCIRI) subsequently aggravates the changes in the microenvironment.^23^ The spinal cord experiences intracellular calcium overload, oxygen-free radical-induced lipid peroxidation, leukocyte activation, inflammatory response, and neuronal apoptosis, which impact and interfere with the stability of the spinal cord microenvironment. SCI caused by compression can progressively cause pathological changes similar to those observed in human patients with SCI, including local hemorrhage, edema, and necrosis followed by partial axonal regeneration and ultimately glial scar and cyst formation; thus, compression SCI models fully meet the needs of researchers. The clamping method allows researchers to induce repeatable and consistent damage. However, the lamina must be removed to achieve compression SCI, which negatively affects the structural integrity of the spine. Another method for modeling compression injury that was described for the first time by Tarlov in 1953 involves the use of a balloon to compress the spinal cord.^24^ In this method, a hole is drilled in the lamina to deliver the balloon to the injury site. Although this method guarantees the stability of the posterior structure of the spine, consistency of the injury site cannot be ensured because the balloon cannot be fixed. Unlike in the contusion model, differences in pathological changes after injury can be quantified by changing the duration of injury and changes in nerve function and metabolism can be assessed in compression models. These models have helped researchers elucidate the role of the glial scar from the perspective of adverse pathological reactions after injury and the promotion of nerve regeneration to explore methods for the treatment of chronic SCI.^25^

Complete or incomplete transaction models are established by using microscopic instruments to transect the exposed spinal cord or remove a small section of the spinal cord, after which tissue engineering materials can be transplanted to alleviate SCI.^26,27^ Because SCI caused by transection is quite different from that observed in patients in hospitals, these models are usually not the first choice of researchers. However, they can minimize the effect of residual nerve fibers in the injury area on the experimental results and allow researchers to effectively observe the pro-regenerative effect of interventional factors on the injured spinal cord. Among modeling methods, transection causes the most severe damage to the spinal cord microenvironment due to induction of local tissue defects. However, these local defects make the implantation of biomaterials possible. Exogenous biomaterials not only provide support for defective tissue but also improve the local inflammatory response and reshape the matrix structure through this unique biochemical properties, thus guaranteeing the continued survival of residual neurons. In addition, biomaterials carrying cells can be transplanted into the injured region. Transplanted cells can repair damaged nerves by promoting axonal regeneration, remyelinate denuded axons, and promote remyelination by endogenous oligodendrocytes.^28^ The beneficial factors released by transplanted cells play a key role in regulating the stability of the microenvironment after injury and ameliorating the inhibition of regeneration post-SCI.^29^ Compared with full transection, which has a high failure rate, hemisection, which is currently more commonly used, substantially reduces the incidence of fatal complications and increases the survival rate because it allows preservation of some neurological function; thus, this method is preferred by researchers.^30^ The dorsal hemisection model is an improved version of the hemisection model. Accurately destroying the corticospinal tract (CST) and the dorsal column, regardless of the strength, speed, and accuracy of the injury, impairs the motor function of the limbs.^31^ This model provides researchers an unparalleled ability to observe axonal regeneration and the effects of tissue engineering materials.

Each SCI model has limitations and advantages, and the present dilemma is that no model completely simulates human SCI, given the complexity of human SCI. Therefore, the functional evaluation criteria for various animal models must be further improved. Improvement of sensory function is also crucial in humans, and the data are encouraging and promising. In addition, while cervical SCI is the most common type of SCI observed in the clinic, thoracic and lumbar segments are usually targeted when establishing animal models due to the advantages of thoracolumbar injury models (high survival rates, ease of operation, and reduced complications). However, we should perform more research on cervical models in the future to obtain more clinically relevant data and allow the translation of research findings. Another point worth noting when selecting a model is the age of the animal. The formation of local scar tissue is one of the main causes of axonal regeneration inhibition after SCI. Some hyperthermic animals, such as fish and amphibians, do not form scar tissue after SCI, allowing axons to regenerate and reconnect quickly. However, adult mammals with SCI may exhibit scar formation that hinders axonal regeneration.^32^ Recent breakthrough research from Zhigang He et al. showed that, unlike in adult mice, the regenerative process in following SCI is not accompanied by scar formation and inflammatory cell accumulation in neonatal (P2) mice; thus, a large number of axons are able to pass through the injury site, allowing regeneration.^33^ Therefore, considering the unique inherent characteristics of the spinal cord at different developmental stages, SCI model animals of different ages should be studied. The current research hotspots and emerging ideas related to SCI require us to consistently develop and improve animal models to ensure that they are standardized, can be quantitatively analyzed, and are relevant, laying a solid foundation for advancing research on the treatment of SCI.

## Pathological mechanisms based on SCI microenvironment imbalance

The pathological mechanisms of traumatic SCI are divided into primary injury and secondary injury. The spinal cord can be injured by cuts, gunshots, lacerations, and blunt injuries. Blood cells flow out from ruptured blood vessels, and injured or dead parenchymal cells release various cytokines. These factors further induce a series of secondary injuries, such as excitotoxicity, neurotransmitter accumulation, free radical production, endogenous opioid expression, cell death, demyelination, axonal degeneration and death.^34^ James W. Rowland et al. defined the stages of SCI as acute (<48 h), subacute (2 d-2 w), intermediate (2 w-6 m), and chronic (>6 m).^35^ In 2018, our team assessed microenvironment imbalance after SCI. Imbalance of the microenvironment at the tissue level, cellular level, and molecular level was observed in the different phases of SCI. In essence, the most critical task associated with nerve repair is remodeling and restoration of intraneuronal balance and extraneuronal balance. Intraneuronal imbalance refers to disorder of intracellular ions, i.e., calcium ions; dysfunction of the cytoskeleton and activation of negative regulatory signaling pathways, which interfere with the normal physiological activity of cells and eventually induce cell death. Similarly, extraneuronal imbalance is mainly manifested as impairment of the secondary inflammatory cascade after SCI, the production of inhibitory extracellular matrix (ECM) and molecules, and disruption of physiological homeostasis, all of which play a dominant role in inducing cell death. Neural regeneration (neuronal replacement, axonal regeneration, and plasticity of neural circuits) and functional recovery are directly related. Thus, we focus on neural regeneration in this review and discuss the relationship between intraneuronal microenvironment balance and the extracellular regenerative microenvironment.

### Intraneuronal microenvironment

#### Neuronal death

After SCI, neurons die immediately due to primary injury. Two hours after SCI, the number of NeuN-positive cells is decreased by 21%. Very few neurons are detected at 24 h, and only a few neurons are detected in the peripheral region of the dorsal horn.^36^ One hour after injury, neuronal apoptosis (DFF40/CAD immunoreactivity) can be detected.^37^ After 3 h, some neurons release cytochrome c, which is an apoptosis indicator^38^ After 4 h, TUNEL-positive cells are found in the gray matter, many of which are neurons. The number of TUNEL-positive cells peaks at 8 h after injury. Only a few apoptotic neurons are observed 24 h after injury. However, DNA fragmentation peaks at 24 h.^39^ Another study showed that necrostatin-1, an inhibitor of necroptosis, reduces acute plasmalemma permeability in neural cells after SCI and reduces the number of PI-positive cells at 24 h after SCI, suggesting a critical role for necroptosis in the early stage after SCI.^40^ Autophagy, or “self-eating”, was first named and observed in the 1960s. This process is complex and includes the initiation of autophagy, the formation of autolysosomes, and degradation.^41^ Autophagy plays a crucial role in SCI. The ratio of LC3-II to LC3-I, which is used to evaluate the degree of autophagy, is significantly increased at 3 d, peaks at 7 d, and is markedly decreased markedly at 21 d after SCI.^42^ However, the function of autophagy in traumatic SCI remains controversial. Some studies have found that autophagy alters presynaptic structures and neurotransmission.^43^ Autophagy may influence regeneration and serve as a crucial factor regulating proper axonal guidance, vesicular release, dendritic spine architecture, spine pruning, and synaptic plasticity.^44–46^ Several well-defined biomarkers, such as Beclin-1, microtubule-associated protein 1 light chain 3B, and p62/SQSTM1, have been used to monitor autophagy following traumatic SCI. Autophagic flux, to which neurons are sensitive, may increase or decrease depending on the location and severity of SCI.^47,48^ Additionally, the type of primary injury may influence autophagic flux.^49,50^ Autophagy and autophagic flux are dynamic after injury and may drive the maintenance of homeostasis and environmental balance. Activation or inhibition of autophagy is advantageous for neuroprotection and has been receiving more attention from researchers in the SCI field. Ferroptosis, an iron-dependent form of nonapoptotic cell death that was identified in 2012, plays an essential role in cell death after SCI.^51^ Ferroptosis-like mitochondrial changes, such as shrunken mitochondria and fewer mitochondrial cristae, are observed at 1 h after SCI. DFO and SRS16–86 significantly increase the expression of a critical protein, GPX4, and enhance neuronal survival, resulting in functional recovery, in a rat spinal cord contusion model.^52,53^ However, further research on the relationship between ferroptosis and SCI is required to explain the detailed mechanism, including the complex relationship between lipid peroxidation and ferroptosis following SCI. Pyroptosis was first observed in macrophages after exposure to *Salmonella* and identified as a unique caspase-1-dependent form of programmed cell death.^54^ The mRNA levels of the genes caspase-1, IL-1β, and IL-18, which are essential for pyroptosis, are elevated one day after SCI, peak at 3 days, and remain at a relatively high level at seven days.^55^

Neurons are the most critical components of the spinal cord, and loss of neurons is the main reason for poor functional recovery after SCI. Reducing secondary neuronal loss or protecting neurons from cell death is the primary goal of SCI treatments. Apoptosis, necroptosis, autophagy, and ferroptosis are related and influence each other to some extent. Necroptosis and ferroptosis may exert detrimental effects, while apoptosis and autophagy may exert beneficial effects in the protection of neurons. The predominant neuronal death pathway after SCI may vary depending on the phase of injury. More research on the correlations between these cell death forms is needed in the future. These efforts may also help improve diagnosis and prognosis.

#### Axonal degeneration and regeneration: an imbalance in the intracellular microenvironment in neurons

After SCI, the number of neurons and axons decreases and reaches the lowest point in the chronic phase. Additionally, the number of myelinated axons decreases significantly. Retrograde axonal degeneration is progressive.^56^ Due to the death of oligodendrocytes, myelin is degraded, aggravating the degeneration of axons. On the other hand, axonal regeneration, a significant process in neural regeneration, is the cornerstone of functional neural circuits. However, axonal regeneration in the spinal cord is poor in mammals following injury. Several factors regulate intrinsic regeneration ability.

- ***The PTEN/mTOR signaling pathway***
  The mammalian target of the rapamycin (mTOR) signaling pathway plays a crucial role in regulating cell growth and proliferation, and the cell cycle. Phosphatase tension homolog (PTEN) is a central negative regulator of the phosphatidylinositol 3-kinase (PI3K)-mammalian target of rapamycin complex 1 (mTORC1) pathway. The mTOR pathway is suppressed in adult retinal ganglion cells after injury. Conditional deletion of PTEN increases mTOR activity to enhance the regeneration of adult corticospinal neurons. Regeneration axons pass through the lesion. PTEN deletion likely intrinsically affects axons rather than survival to promote axonal regeneration after injury.^57,58^ AKT inhibition significantly reduces PTEN deletion-induced axonal regeneration, and PTEN deletion results in AKT activation to a minor extent, causing adequate mTORC1 activation to allow axonal regrowth.^59^ The results of other experiments have indicated that mTORC1 activation and GSK3β inhibition act in parallel and synergistically downstream of AKT to promote axonal regeneration in the CNS.^60,61^ AKT-dependent and AKT-independent pathways work together and cooperate closely in PTEN deletion-induced axonal regeneration in the CNS. Additionally, some interesting studies have indicated that IL6, exercise, and electrical stimulation activate the mTOR signaling pathway, promoting axonal outgrowth.^62–64^
- ***Cytoskeletal dynamics***
  The cytoskeleton of neurons is mainly composed of actin filaments, which maintain a highly polarized shape and extension in the proper direction, and microtubules, which alter the structure of the axon. The formation of new growth cones mediated by the regular dynamic assembly and transport of the cytoskeleton is a prerequisite for axonal regeneration.^65^ However, following SCI, central axons present a swollen and dystrophic growth-incompetent structure, namely, the retraction bulb, which is presumed to be the cause of the weak regenerative capacity of the CNS.^66^ Injured axons gradually retract from 1 to 35 days after injury and eventually form a disorganized ending.^67^ During this process, the ratio of stable detyrosinated microtubules and dynamic tyrosinated microtubules increases. Stable microtubules are markedly disassembled, mainly manifesting as disordered accumulation in the growth cone center. In recent years, the important role of glycogen synthase kinase 3 (GSK3) and Rho GTPase in the dynamic regulation of microtubules has received increasing attention.^68^ GSK3, a downstream effector of PI3K, phosphorylates proteins involved in regulating microtubule-based transport and microtubule dynamics.^69,70^ Small GTPases of the RHO family, including rho, cdc43, and rac, are responsible for promoting the interaction between actin and microtubules in neurons, which helps regulate the neuronal cytoskeleton, especially actin filament dynamics, after SCI.^71,72^
- ***Energy supply***
  Reconstruction of the cytoskeleton, transportation of synthetic materials, and assembly of axon components are critical processes involved in axonal regeneration that undoubtedly require energy.^73^ Creatine, a substance that produces ATP in animals, promotes the regeneration of CST axons, indicating that the energy supply contributes to axonal growth after SCI.^74^ In cells of higher animals, oxidative phosphorylation in mitochondria provides approximately 95% of total ATP.^75^ The ATP content begins to decrease 2 h after SCI, and over time, ATP production gradually decreases.^76^ Sun Min Han et al. showed that the mitochondrial density in injured axons is increased at 6 h.^77^ Axonal disruption can lead to acute mitochondrial depolarization and ATP depletion in injured axons. A decrease in mitochondrial motility and lack of energy in injured axons are the internal mechanisms controlling the regeneration of mature neurons.^78^ Accordingly, an increase in the aggregation of mitochondria at the end of axons potentially accelerates axonal regeneration.^79^ More precisely, normal axonal regeneration requires proper mitochondrial respiratory chain function.^77^
  Many factors are involved in energy production by mitochondria. For example, dual leucine zipper kinase 1 (DLK-1) and microtubule-based kinesin and helper proteins (such as the mitochondrial protein Miro and adapter Milton) participate in the transport and localization of mitochondria in axons after nerve injury, increase energy production and promote axonal growth.^77,80^ In addition, loss of function of nuo-6 and isp-1, which encode different mitochondrial respiratory chain subunits, reduces energy production,^81,82^ ultimately decreasing nerve regeneration.^77^ Deletion of the snph gene enhances mitochondrial transport activity in axons, reverses the mitochondrial damage caused by SCI, and promotes the growth of axons and the formation of synaptic connections, thus improving motor function.^74^ In the future, treatments that improve bioenergy metabolism to coordinate the recovery of the energy supply may represent new therapeutic strategies and research directions for promoting axonal regeneration and function after central nervous system (CNS) injury or other neurological diseases.^74,83^

### Regeneration microenvironment imbalance

#### Glial scar and fibrotic scar (Fig. 2)

- ***Astrocytes***
  Astrocytes are organic components of the CNS that are distributed throughout the CNS and perform many functions essential for normal neuronal development, synapse formation, neural circuit function, and propagation of action potentials.^84–86^ They provide neurons with energy and neurotransmitters to maintain CNS homeostasis and act as a physical barrier between the synaptic connections of adjacent neurons.^87^ There are different subtypes of astrocytes. Neuroinflammation and ischemia induce two the polarization of reactive astrocytes into the A1 and A2 phenotypes.^88^ A1 astrocytes upregulate the expression of complement cascade genes that destroy synapses and are harmful to the nervous system. In contrast, A2 astrocytes upregulate the expression of various neurotrophic factors, which may exert a protective effect on nerves.^89^ These astrocytes eventually become scar-forming astrocytes, which prevent axonal regeneration and nerve repair through a process known as reactive astrogliosis.^90^

**Fig. 2:**
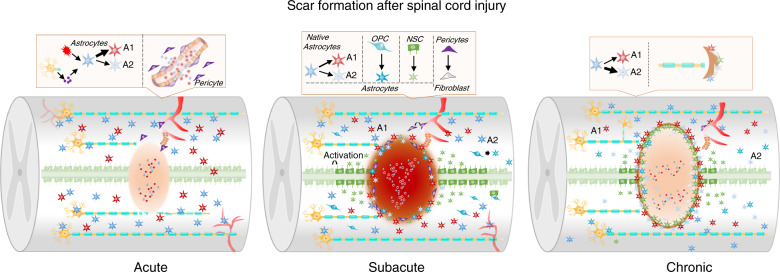
Scar formation after SCI. This figure shows glial scar and fibrotic scar changes in the acute, subacute, and chronic phases. In the acute phase of SCI, astrocytes are polarized toward the A1 and A2 phenotypes, and pericytes derived from blood vessels migrate into the injury epicenter. In the subacute phase of SCI, a scar is formed by astrocytes derived from native astrocytes, oligodendrocyte progenitor cells (OPCs) and neural stem cells (NSCs). In this stage, fibroblast-derived pericytes seal the scar. In the chronic phase of SCI, the scar is stable, limits inflammation and suppresses the regeneration of axons
  In the scar formation stage, type I collagen is expressed at high levels in the spinal cord and induces astrocytic scar formation through the integrin-N-cadherin pathway to prevent axonal regrowth.^91^ Chondroitin sulfate proteoglycans (CSPGs) produced by astrocyte scars are considered the leading cause of regeneration failure. They effectively preventing regenerating axons from crossing the SCI lesion.^92^ A variety of cells (including pericytes, fibroblast lineage cells, and inflammatory cells) produce CSPGs after SCI.^93^ Moreover, CSPGs and their receptors PTPσ and NgR regulate development and synaptic plasticity in adulthood.^94^ Therefore, it is unclear whether CSPGs produced by the astrocytic scar are the main factors underlying the inability of axons to regenerate across the SCI lesion.^95,96^ Astrogliosis might exacerbate the inflammatory response after trauma or autoimmune attack.^97^ However, transgenic ablation or prevention of astrocyte hyperplasia or astrocyte astrocytic formation increases inflammation and tissue damage and worsens functional recovery.^98,99^ Prevention of astroglial scar formation fails to promote injured axon regeneration, indicating that astroglial scars may contribute to CNS regeneration.^100^ Therefore, as the astrocytic scar limits inflammation and protects adjacent healthy tissues, the potentially harmful effects of the astrocytic scar should be alleviated rather than preventing or eliminating the astrocytic scar.^101^
- ***Pericytes***
  Pericytes have long protrusions that surround the blood vessel wall and participate in blood flow regulation, blood vessel development, maturation, and remodeling^102,103^ and cooperate with astrocytes to regulate the integrity of blood–brain barrier function.^104^ Scholars have proposed that reactive astrocytes are critical for inhibiting axonal regeneration. However, experts have also postulated that the pericyte-derived fibrous scar is the main obstacle to axonal regeneration. Moderate inhibition of this process causes complete wound healing and inhibits inflammation and reactive astrogliosis while allowing axonal regeneration and improving functional recovery.^100,105,106^ During the process of fibrous scar formation in various organs and pathological tissues, ECM proteins are deposited by a large number of fibroblasts to form connective tissue.^107^ Following SCI, the most obvious features of the fibrous scar are the accumulation of fibroblasts around blood vessels and the deposition of the ECM protein fibronectin.^108^ Furthermore, fibroblasts are the most likely primary source of fibronectin in the fibrous scar. Type A pericytes, which express glutamate aspartate transporter (Glast), leave the blood vessel wall after SCI and form fibroblast-like cells that promote scar tissue matrix component deposition. These scar-forming pericytes account for approximately 10% of all pericytes.^106^ Glast-rasless mice exhibit reductions in inflammation and the number of astrocytes after SCI in addition to decreased scar formation and promotion of axonal regeneration in the CST^.109^ However, loss of proliferative NG2^+^ pericytes after SCI prevents blood vessel formation in the lesion.^110^ Moreover, pericytes have a critical ability to regulate capillary tension and spinal cord blood flow and may be involved in neuropathic pain, inflammation, myelin, neural regeneration and astrocyte regulation.^111–115^ Due to the wide diversity of pericytes and the differences in the functions of different pericyte subtypes after SCI, a targeted strategy is needed.

#### The role of microglia and macrophages in inflammation after SCI (Fig. 3)

Microglia are immune cells in the CNS that assess whether the CNS is damaged or infected, engulf dead cells and cell debris, and participate in synaptic remodeling during neural development. Microglia are activated after SCI, but whether they are useful or harmful remain controversial. Activated microglia release proinflammatory factors and cause secondary damage,^116^ but they can also exert beneficial effects include by inhibiting lesion expansion, removing debris, and producing anti-inflammatory factors.^117,118^ Microglia are activated rapidly after acute SCI, and the dense cytoplasmic network surrounding the lesion helps to inhibit the expansion of the lesion.^117^ The rapid response of microglia to injury is mediated by the binding of purinergic receptor (P2Y12R) to ATP released by damaged cells or astrocytes.^119^ The activation of microglia is similar to that of macrophages, as they become polarized. Polarized microglia are divided into two types: classically activated (M1) microglia, which are typically activated by IFNγ and LPS, and alternatively activated (M2) microglia, which can be further subdivided into M2a (activated by IL-4 or IL-13), M2b (immune complexes that bind to IL-1beta or LPS) and M2c (respond to IL-10, TGFbeta or glucocorticoids) microglia.^120^ However, the characteristics of these activated microglia are currently controversial. Some experts suggest adopting a related naming method that reflects the stimulus that induces activation, i.e., M (IL-4), M (Ig), M (IL-10), M (LPS), etc.^121^. The phenotypic and antigenic similarity between microglia and macrophages makes it difficult to distinguish the two populations. Flow cytometry can be used to distinguish CD45hi/CD11b^+^ macrophages from CD45lo/CD11b^+^ microglia, but this method does not provide information on the spatial distribution of the cells. Many researchers have measured the expression levels of certain antigens to distinguish the two populations. For example, spinal cord microglia express the chemokine receptor Cx3Cr1 at a much higher level than macrophages, and the expression levels of CD45 and Mac-2 (galectin-3) in macrophages are higher than those in microglia.^122^ Resident microglia activate and contact damaged axons at 0–2 days after SCI, while macrophages are the main cells that mediate phagocytosis 3 days after injury. Afterward, infiltrating macrophages rather than microglia become the main cells that contact degenerated axons and contain more phagocytic material, and this phenomenon persists for up to 42 days.^123^ Resident microglia form a boundary around the lesion that prevents damage from spreading. In contrast, bone marrow-derived macrophages enter the lesion epicenter, engulf apoptotic and necrotic cells, and clear tissue fragments after SCI.^116^

**Fig. 3:**
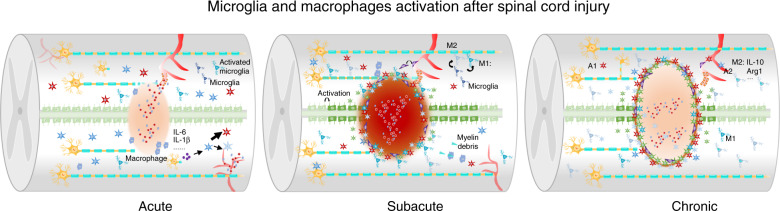
Microglial and macrophage activation after SCI. This figure shows the changes in microglia and macrophages after SCI. In the acute phase of SCI, microglia are activated by cytokines and factors released from injured neural cells. Macrophages from blood vessels infiltrate injured tissue. In the subacute phase of SCI, M1 microglia and macrophages dominate and exacerbate inflammation by releasing inflammatory factors. Activated microglia and macrophages swallow injured or dead neural cells and myelin. In the chronic phase of SCI, microglia are mainly M2 microglia, which promote regeneration

After microglial activation, released chemokines attract many blood-borne immune cells that penetrate the damaged blood spinal cord barrier. Blood-derived monocytes differentiate into macrophages and, together with microglia, form an innate immune defense; however, they also release inflammatory cytokines that aggravate secondary damage.^117,124^ Many previous studies have explored the M1/M2 polarization of microglia. Reactive oxygen and nitrogen species (ROS and RNS, respectively) and lipid peroxidation mediate M1 macrophage activation and the M2 to M1 transition.^125,126^ Transcriptional events might regulate the macrophage phenotype. NF-κB, STAT1, and interferon regulatory factor 5 drive proinflammatory M1 macrophage polarization. In contrast, the transcription factors that regulate M2 macrophage polarization include STAT6, IRF4, and peroxisome proliferator-activated receptors.^127^

Activated microglia can produce various proinflammatory cytokines, proteases, and other cytotoxic factors after SCI. At 3 days postinjury, IL-1β and TNF-α expression peaks, leading to the apoptosis of neurons and oligodendrocytes.^128,129^ According to recent studies, microglia/macrophages play essential roles in neurogenesis, axonal regeneration, synaptic plasticity, angiogenesis, and vascular repair. Triggering receptor expressed on myeloid cells 2 (TREM2), a member of the TREM family of innate immune receptors, is expressed on microglia. Stimulation of TREM2 on microglia increases phagocytosis and reduces the expression of proinflammatory cytokines, while TREM2 knockdown impairs the phagocytosis of apoptotic neurons and increases the expression of TNFα and iNOS.^116,130^ Microglia that express IL-10 inhibit inflammation and promote axonal regeneration.^131^ When microglia are activated, the ECM component fibronectin, the level of which is transiently increased, bridges the two spinal cord stumps and promotes axonal regeneration.^33^

#### Demyelination and remyelination (Fig. 4)

##### Loss of oligodendrocytes (demyelination)

In the acute phase of SCI, death of oligodendrocytes is mediated by apoptosis, necrosis, and autophagy. Apoptosis of oligodendrocytes occurs 6 h after SCI in rats and lasts for up to 3 weeks in monkeys.^132^ Oligodendrocyte apoptosis leads to the demyelination of axons after SCI.^133,134^ Oligodendrocyte necrosis occurs within 24 h after SCI and induces inflammation.^135,136^ An imbalance in the microenvironment after SCI causes the death of oligodendrocytes. After SCI, the concentration of glutamate increases six-fold.^137^ In the CNS, oligodendrocytes express glutamate receptors and are the targets of glutamate toxicity.^138^ Glutamate induces a decrease in GSH levels in oligodendrocytes and leads to cell death associated with excessive ROS production.^139^ After SCI, the concentration of extracellular iron increases rapidly.^140^ Our previous study also found that iron levels begin to increase at 1 h and remain at a high level at 24 h after SCI.^52^

**Fig. 4:**
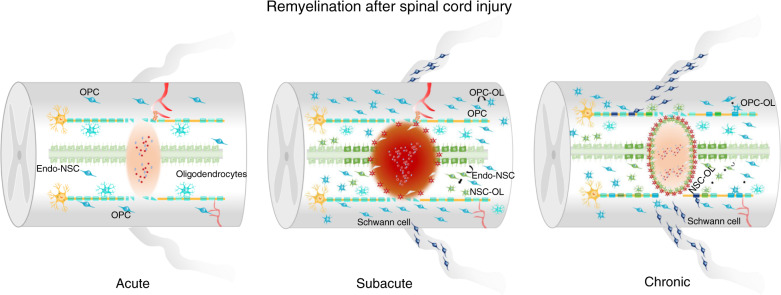
Remyelination after SCI. This figure shows the changes in remyelination after SCI. In the acute phase of SCI, the number of oligodendrocytes is reduced, and the integrity of myelin is disrupted. In the subacute phase of SCI, OPCs are activated and begin to differentiate into new oligodendrocytes. Additionally, a small number of OPCs can differentiates into Schwann cells. endo-NSCs are activated, and some of them differentiate into oligodendrocytes. In the chronic phase of SCI, newborn oligodendrocytes form myelin around spared or regenerated axons

Fe^2+^ in cells generates many ROS through the Fenton reaction,^141^ which induces lipid peroxidation and ultimately leads to ferroptosis. Iron-loaded cells rapidly die after incubation with TNFα and IFN.^142^ As shown in our previous study, OLN93 oligodendrocytes undergo ferroptosis and exhibit inhibition of glutathione peroxidase 4 expression.^143^ Inflammatory factors also induce the apoptosis of oligodendrocytes. TNFα induces oligodendrocyte apoptosis through its interaction with TNFR1 and TNFR2, further leading to myelin degradation.^144–147^ Precursors of neurotrophic factors also induce oligodendrocyte death.^148^

Some studies have shown that autophagy occurs in oligodendrocytes after SCI.^149,150^ However, autophagy does not lead to the loss of oligodendrocytes after SCI.^151^ Bankston et al. reported that Atg5^−/−^ oligodendrocytes do not form normal myelin.^152^ Additionally, Saraswat Ohri et al. found that depleting Atg5 from oligodendrocytes blocks autophagy and further limits functional recovery after SCI.^153^

Following the death of oligodendrocytes and myelin breakdown, the microenvironment is not conducive to axonal regeneration. Myelin-associated inhibitors include Nogo, oligodendrocyte myelin glycoprotein (OMgp), and myelin-associated glycoprotein (MAG). Three isoforms of Nogo proteins have been identified, namely, Nogo-A, Nogo-B, and Nogo-C; Nogo-A exerts the strongest inhibitory effect and is expressed at higher levels in oligodendrocytes than in Schwann cells (SCs).^154–156^ The Nogo-66 domain of the protein interacts with receptors or other protein partners, such as paired immunoglobulin-like receptor B, Nogo receptor 1, and Nogo receptor 2, to inhibit axon regeneration or lead to growth cone collapse.^157^ Additionally, MAG can interact with the Nogo receptor,^158^ but it binds with higher affinity to NgR 2.^159,160^ At three days postinjury, increased expression of TGFβ, CNTF, and FGF-2 promotes the migration and differentiation of OPCs,^161,162^ and remyelination occurs in the spinal cord. However, during repair of the injured spinal cord, NG2^+^ OPCs secrete CSPGs, which inhibit growth. NG2 may help prevent axonal death but also limits axon extension. Furthermore, NG2 is cleaved by matrix metalloproteinases (MMPs) and released into the extracellular matrix (ECM) to inhibit axonal growth.^163–165^

##### Oligodendrocyte replacement (remyelination)

- ***Oligodendrocyte progenitor cells***
  Oligodendrocyte progenitor cells (OPCs) are the primary sources of oligodendrocytes for remyelination after SCI. The number of BrdU/Olig2^+^ OPCs in the injury epicenter is decreased by approximately 50% at 24 h after SCI,^166^ whereas the total number of NG2^+^ cells in the epicenter does not change and the density of NG2^+^ cells increases slightly from 2 days to 42 days.^167^ However, the number of NG2^+^ cells at the lesion border is increased significantly at 2 days, and the proliferation of OPCs peaks at approximately two weeks.^168^ Thus, in the injury epicenter, the number of OPCs decreases within 24 h, and then OPCs are activated and rapidly proliferate, migrate, and differentiate into mature oligodendrocytes. However, NG2 is expressed not only in OPCs but also in pericytes. Thus, more work is needed to map the fate of OPCs from the acute phase (especially within 24 h) to the chronic phase. A recent study using fate mapping showed that ~30% of OPCs give rise to oligodendrocytes by 12 weeks after SCI.^169^ However, myelin is not well restored after SCI, and factors that disrupt the microenvironmental balance, such as myelin debris, and the activation of the MBP signaling pathway and proinflammatory factors, are the main factors that inhibit the differentiation of OPCs^170^ and the maturation of new oligodendrocytes.
  However, one study reported that locomotor recovery after SCI does not require oligodendrocyte remyelination. The authors found that knocking out the myelin regulatory factor *Myrf* in OPCs decreased the number of new oligodendrocytes after SCI. Consequently, the number of myelinated axons was reduced by 44% in the lesion epicenter. However, no difference in locomotor recovery was observed between the two groups.^171^ Some new oligodendrocytes were observed in the injury epicenter in the knockout group, and these cells contributed to the recovery of locomotor function to some extent. Additionally, surviving adult oligodendrocytes participate in remyelination in some CNS disease animal models.^172^ Thus, more research is needed to determine the role of new and surviving oligodendrocytes in SCI.

- ***Schwann cells***
  Many studies have reported that SCs are present after SCI.^173–175^ According to some studies, the SCs observed after SCI are derived from nerve roots.^176,177^ Nagoshi et al. used transgenic mouse lines and showed that after SCI, EFGP^+^ -derived mature SCs (P0^+^) residing at nerve roots dedifferentiated into immature SCs (P0^−^/P75^+^/c-Jun), which infiltrated the spinal cord lesion.^176^ Additionally, Assinck et al. used P0 Cre-ER:YFP mice to examine the contribution of SCs from peripheral nerves after SCI and found that only a very small amount of new SCs (<10%) were derived from the peripheral nerve, whereas approximately 70%–80% SCs were derived from resident OPCs (without considering the recombination efficiency).^169^ However, some issues should be considered. Which type of axons are remyelinated by SCs? Emerging SCs were distributed in the dorsal columns. What is the contribution of new SCs to functional recovery after SCI? What are the critical factors in the microenvironment that induce OPC differentiation into SCs?

- ***Endo-NSCs***
  Endogenous neural stem cells (Endo-NSCs), which reside in the central canal of the spinal cord, self-renew and differentiate into different neural cell types. Endo-NSCs remain inactive in normal physiological environments but are activated by conditions such as SCI.^178,179^ Following SCI, Endo-NSCs progress through three crucial steps: activation, migration, and differentiation.^180^ However, the ability of Endo-NSCs to differentiate into neurons is limited. Barnabé-Heider et al. found that ependymal cells (FoxJ1-CreER-recombined cells) give rise to astrocytes (Sox9^+^ and GFAP^+^) and some oligodendrocytes (Olig2^+^ and APC^+^). However, no neurons are derived from FoxJ1-CreER recombined cells.^181^ This phenomenon may be associated with the high expression of Notch1 and Hes1, which likely inhibit neuronal differentiation after injury.^182,183^ Recently, Llorens-Bobadilla et al. identified a latent linage of endo-NSCs that can be used in oligodendrocyte replacement.^184^ OLIG2-overexpressing adult ependymal cells efficiently differentiate into oligodendrocytes after SCI.

#### Interneurons: their vital role in neural circuit reconstruction

Spinal cord interneurons are located in the spinal cord and project between different spinal cord segments.^185^ Spinal interneurons are classified into several subtypes according to various factors. According to location, these interneurons can be divided into dorsal interneurons (dI1–6) and ventral interneurons (V0-V3, VX).^186^ According to the length of their axonal projections, they can be divided into short interneurons, which project between several spinal cord segments (e.g., between cervical or thoracic spinal cord segments), and long interneurons, which project between many segments (e.g., from the cervical spinal cord to the thoracic spinal cord). According to whether their projections cross the spinal cord midline, spinal cord interneurons can be divided into ipsilateral and commissural interneurons. According to the type of neurotransmitter, spinal cord interneurons can be divided into glutamatergic excitatory (e.g., V0v, V2a, V3, etc.) interneurons and GABA/glycinergic inhibitory (V0D, V2b, dI6, etc.) interneurons.^185,187^

Many experiments have proven that spinal cord interneurons can form new paths to promote functional recovery.^188,189^ The formation of these new paths is mainly achieved through the three mechanisms described below.

##### Receiving of collateral sprouts

Spinal cord interneurons receive collateral sprouts from neurons in the spinal cord to form new synaptic connections. Some researchers have confirmed that interneurons in rats with SCI receive collaterals from the CST and the reticulospinal tract to form new synapses, which ultimately contribute to the restoration of function.^190–192^ Liu et al. showed that V2a interneurons receive undamaged sprouting axons from the CST across the midline after treatment with docosahexaenoic acid, ultimately resulting in improvement of proximal function associated with the injured area.^193^ These experimental results prove that new connections formed by interneurons and other neurons in the spinal cord circuit after SCI are essential for spinal cord function recovery.

##### Formation of a bypass pathway

Interneurons near the lesion level can be recruited to form a relay circuit that may bypass the injury epicenter after SCI.^194^ Courtine et al. found that a retrograde tracer can be transported from below the injury site to above the injury site in a T12 and T7 cross-hemisection rat model. The results prove that propriospinal neurons bypass the injury site, leading to spontaneous functional recovery. The reorganization of descending and propriospinal neurons also promotes functional recovery in subjects with severe SCI.^195^ Based on these results, reconnection of descending inputs and propriospinal circuits is sufficient to achieve functional recovery.

##### Participation in sensory feedback

Sensory feedback is essential for functional recovery after complete SCI,^196^ and interneurons are involved in this process. For example, dI3 interneurons are participate in short-term modulation of movement but are not required for normal walking. Bui et al. abolished dI3 interneuron synaptic transmission through genetic methods in mice. Functional recovery was impaired in SCI mice compared with control mice due to the “removal” of dI3 interneurons.^197^ Another study showed that sensory feedback and a brain-computer interface of residual tactile signals in the hands can improve the sensory and motor functions of paralyzed muscles in patients with clinically complete SCI.^198^ Therefore, sensory feedback via interneurons can promote functional recovery after SCI.

Although interneurons have been identified as beneficial for repair of the injured spinal cord, some problems remain to be addressed. (1). Unclassified subgroups of interneurons need to be characterized. Although more than 20 subtypes of spinal cord interneurons (spins) have been identified according to their location, electrophysiological characteristics, and specific transcription factors,^186^ more subtypes should be identified.^185^ (2). The complex functions and interactions of interneurons need to be explored. On the one hand, interneurons at different spinal cord levels are diverse. Homeodomain transcription factors can be used to identify distinct interneuron populations, but the expression of transcription factors varies among spinal cord segments.^199^ These differences may affect our understanding of motor networks at the cervical and lumbar levels.^185^ On the other hand, diverse interneurons may interact with each other. Shox2+ non-V2a and VX cells are related to rhythm production. However, Shox2+ non-V2a and VX spinal interneurons are not the only neurons that produce rhythms, and thus, the relationship between these neurons and other neurons that produce rhythms should be considered in future studies.^200,201^ These complex issues undoubtedly increases the technical requirements for interneuron studies. 3). The effects of interneurons are unclear. Although spinal interneurons have been proven to be involved in anatomical reorganization after SCI,^202^ this reorganization may not be adaptive (e.g., it may result in neuropathic pain).^203^ A nonadaptive increase in connections between inhibitory interneurons and spinal motor networks may limit motor recovery after SCI.^204^ Additionally, overrecruitment of excitatory interneurons into the sensory network may increase pain or spasm. An increase in excitatory inputs to inferior motor neurons without regulation of inhibitory spinal neurons may also lead to hyperactivity, neuronal damage, and functional loss.^205^

## Repair strategies and translational progress

### Pharmacological repair strategies for spinal cord injury

**Fig. 5:**
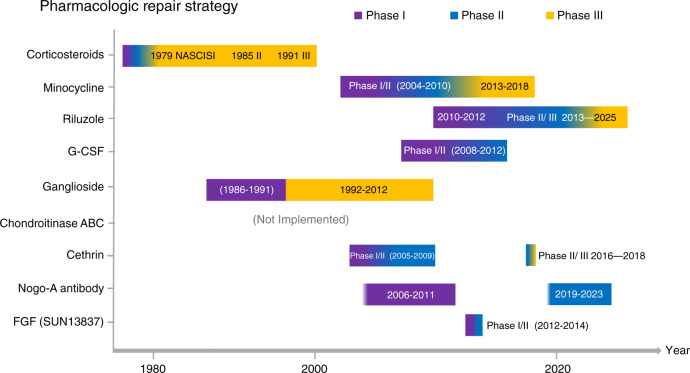
Research progress on pharmacological strategies for repairing the injured spinal cord. This figure shows clinical trials on medicines for the treatment of SCI, including corticosteroids, minocycline, riluzole, G-CSF, ganglioside, chondroitinase ABC, cethrin, Nogo-A antibodies, and FGF

The microenvironment must be regulated after local injury, and intrinsic regenerative processes must be activated to promote repair of the injured spinal cord. A large number of promising new medicines are emerging.^73,206^ Potential medications that regulate the injured microenvironment and promote neuroprotection have attracted increasing attention (Fig. 5, Table 1).

**Table 1:** Pharmacologic repair strategy of spinal cord injury

Medicine	Mechanism	Representative findings
Microenvironmental regulator		
Corticosteroids	• Upregulate the release of anti-inflammatory cytokines • Reduce the extravasation of inflammatory cells • Promote the survival of neurons	• NASCIS-1, NASCIS-2 and NASCIS-3 has been completed. • The controversial research is still being further clarified.
Minocycline	• Excellent lipid solubility^206^ • Reduce inflammatory response and prevent neuronal apoptosis^207^	• Phase II study proved that minocycline can significantly improve the ASIA motor score for patients with SCI.^208^ • Phase III is underway
Riluzole	• Sodium channel blocker • Inhibiting excitotoxicity by reducing the release of presynaptic glutamate to inhibit excitotoxicity^209^	• Phase I trial showed that Riluzole is effective in improving the ASIA score without serious adverse events • Phase II/III multicenter, randomized trial is in progress^210,211^
Granulocyte-colony- stimulating factor (G-CSF)	• Promote differentiation and proliferation of granulocytes • Induce migration of bone marrow mesenchymal cells to SCI sites and inhibit apoptosis^212^	• Phase I/II trial have shown that CSF can significantly improve motor function in patients with cervical and thoracic spinal cord injuries for 5 days.^213^
Chondroitinase ABC (ChABC)	• Eliminate CSPG glycosaminoglycan (GAG) chains to achieve the inactivation of CSPGs^94^ • Promoting axonal regeneration	• Preclinical researches have shown that the superiority of ChABC in regulating the inhibitory environment^214–216^
Ganglioside (GM-1)	• Downregulate caspase-3 and upregulate the expression of NGF • Maintain neuronal cell survival	• Phase I clinical trial conducted a preliminary assessment of the safety and effectiveness of GM-1 • Phase III trial shown that GM-1 did not show a significant difference in the improvement of the primary outcome measures. • More experiments are demonstrating the role of GM-1 in spinal cord injury.
Magnesium	• Block NMDA receptors to prevent glutamatergic excitotoxicity	• Preclinical studies have demonstrated that Magnesium has a neuroprotective effect on the rat model of SCI and optimizes motor functional outcome^217^
Neuroregenertive activator		
Cethrin	• A recombinant of VX-210 • Involve the down-regulation of the RHOA pathway to promote axonal regeneration^218^	• The phase I/IIa study has demonstrated the effectiveness of this medicine in improving motor function^219^ • Phase IIb/III trial is being carried out^220^
Nogo-A antibodies	• The antibody of a myelin-derived axon growth inhibitor • Inhibit Nogo-A to facilitate nerve repair and reconstruction after SCI	• Nogo-A antibodies have proved an attraction in facilitating nerve regeneration in preclinical SCI trials, and a phase I research has been completed^221,222^ • Phase II placebo-controlled trial is underway
Basic fibroblast growth factor (bFGF)	• A ligand of tyrosine kinase receptor • Inhibit inflammatory response, glial scar and astrogliosis and stimulate axon regeneration • Neuroprotective function	• A preliminary Phase I clinical study has shown that FGF may have a positive effect in improving ASIA motor score • Phase I/II trial has evaluated with the unpublished conclusion^223,224^

#### Microenvironment-regulating medicines

##### Corticosteroids

A large amount of evidence from preclinical studies has revealed the effect of MP in improving the microenvironment after SCI. The main effect of MP is regulation of neuroinflammation after SCI. As a key mechanism underlying the progression of SCI, neuroinflammation increases the migration, activation, and differentiation of leukocytes at the injury site. Researchers have found that MP increases the release of anti-inflammatory cytokines, reduces the extravasation of inflammatory cells, promotes the survival of neurons, and further alleviates the inflammatory microenvironment induces by SCI.^226,227^ The most significant controversial issue related to the pharmacological treatment of SCI is whether high-dose intravenous methylprednisolone (MP) is required in the acute phase. In preclinical evaluations, researchers found that MP increases the release of anti-inflammatory cytokines, reduces oxidative stress, and improves neuronal survival.^226,227^ Three large-scale clinical randomized controlled studies named the National Acute Spinal Cord Injury Studies (NASCISs) have been conducted to evaluate the effectiveness of MP in the treatment of SCI. The first NASCIS involved 330 patients with acute SCI who were administered 1000 mg of MP. The results showed no significant differences in neurological recovery of pinprick and light touch sensation or motor function at six weeks or six months after injury.^228^ Subsequently, in NASCIS-2, a 5-point increase in the ASIA exercise score was observed in the MP group compared to the placebo group at the 6-month follow-up.^229^ However, flaws in methodology, science, and related factors, such as an insufficient sample size and lack of functional outcome measures, have prevented this research from being highly recognized by peers.^230,231^ In NASCIS-3, it was found that extending MP therapy beyond 24 h for patients who were administered MP within 3 h of injury did not result in a favorable outcome. However, patients with SCI who receive MP treatment 3–8 h after injury showed satisfactory motor function improvement when MP administration was continued for 48 h.^232^

In 2012, a systematic review and meta-analysis using Cochrane methods that included six randomized observational studies revealed that the ASIA exercise score of patients receiving MP treatment within 8 h after injury increased by 4 points.^233^ The 2017 AO Spine guidelines suggest that MP should be administered to patients for 24 h within 8 h of SCI.^234^ However, in a cohort study from Canada in 2015, the authors found that the administration of MP did not effectively improve the motor function of patients with SCI and caused some adverse effects, such as urinary tract and pulmonary infections.^235^ A recent meta-analysis by Liu et al. showed no significant difference in the improvement of motor and sensory scores after SCI between the MP group and the control group. Since some adverse events occur after MP administration, the (authors recommend against using this corticosteroid in the early stage after SCI.^236^ The latest guidelines of the French Society of Anesthesia and Intensive Care Medicine (the 2020 guidelines) state that early administration of steroids is strongly not recommended for patients with SCI, arguing that the treatment does not improve neurological prognosis.^237,238^

Thus, several problems need to be addressed before MP can be safely and effectively applied in the clinic. 1) More scientific research on the optimal time window of MP administration to effectively prevent secondary injury after SCI should be conducted. (2) The ideal dosage and administration route, such as intravenous or local administration, should also be determined. (3) Differences in the effect of MP on different SCI types and degrees and individual differences should be fully considered before determining whether to use MP after SCI to prevent unexpected complications and achieve the greatest therapeutic benefit.

##### Minocycline

Minocycline is a tetracyclic antibiotic with excellent lipid solubility. Its effects on SCI were first reported by Wells et al. in 2003. Minocycline can ameliorate the local inhibitory microenvironment following SCI,^207,239^ mainly by attenuating cell death and reducing the levels of free radicals, inflammatory cytokines, and MMPs.^208^ SCI is often accompanied by activation of multiple cell death signals, which induce the death of neurons and oligodendrocytes. Minocycline treatment not only resists the inflammatory microenvironment by increasing interleukin-10 expression and reducing tumor necrosis factor-alpha expression but also inhibits the further development of apoptosis after injury by significantly decreasing caspase-3 activity. The positive results observed in SCI models, such as satisfactory functional overcomes in the inclined plane test and good BBB scale scores, neuroanatomic reorganization, and improvements in the molecular environment, indicate that minocycline has great potential to be translated to the clinic for SCI treatment in the future.^240,241^

In 2003, Wells et al. first reported that minocycline ameliorates the local inhibitory microenvironment following SCI,^207,239^ mainly by promoting oligodendrocyte survival and reducing the levels of free radicals, inflammatory cytokines, and MMPs.^208^ Some positive results have been observed in SCI models, such as satisfactory functional overcomes in the inclined plane test and good BBB scale scores, neuroanatomical reorganization, and improvements in the molecular environment, showing the potential for the future translation of this medicine.^240,241^ A phase I/II clinical trial (NCT00559494) involving dose optimization, safety assessments, and outcome effectiveness evaluations for minocycline was completed in 2010. Although the administered dose of minocycline was higher than the previously reported maximum dose of minocycline used in humans, the minocycline levels in the serum and cerebrospinal fluid were at steady-state concentrations. In addition, only one patient in the drug intervention group presented elevated liver enzyme levels. Compared with the placebo group, the minocycline intervention group showed improvements in motor and sensory function.^209^ Minocycline was also studied in a large-scale multicenter phase III clinical trial (NCT01828203). However, several recent studies on the efficacy of minocycline for SCI have produced results that are inconsistent with those of previous promising studies, which adds uncertainty to the future translation and development of this medicine.^242,243^

##### Riluzole

Riluzole is a sodium channel blocker approved by the FDA to prevent the progression of amyotrophic lateral sclerosis. Significant manifestations of the microenvironmental imbalance after SCI are changes in the physical and chemical properties of functional nerve cells at the injury region, such as continuous activation of neuronal voltage-gated sodium ion channels. Due to the progression of cell swelling, glutamine excitotoxicity, and acidosis, these detrimental effects eventually lead to an increase in cell mortality. Some encouraging studies have reported that riluzole can inhibit the sodium-dependent glutamatergic system and can alleviate neurological dysfunction and improve neuroelectrophysiological and behavioral overcomes after SCI.^210,244^ A prospective multicenter phase I clinical trial showed that the administration of riluzole in the early stage of SCI significantly improves the ASIA score without causing serious adverse events.^211^ In addition, a recent systematic review found that riluzole significantly improves motor scores and gait function in preclinical models of traumatic and nontraumatic SCI, providing important supplemental data to clinical studies assessing the effect of riluzole on traumatic and nontraumatic SCI.^212^ Nevertheless, some unexpected side effects, such as locomotor ataxia and lethargy, occurred in the high-dose administration experiment,^245^ which poses a new challenge in determining the appropriate dosage for future clinical translation.

##### Granulocyte colony-stimulating factor (G-CSF)

G-CSF, which stimulates granulocyte migration and proliferation, is used primarily to treat neutropenia and after transplantation. The effect of G-CSF in improving the microenvironment after injury is reflected by its ability to mobilize bone marrow-derived stem cells to reconstruct disrupted neural structures. G-CSF increases the proliferation of neural stem cells (NSCs), the recruitment of NSCs and their progeny to the injury site, and angiogenesis, which creates favorable conditions for reconstruction. Additionally, the immunomodulatory and anti-apoptotic effects of G-CSF allow it to protect residual nerve cells in the injury epicenter^213^ Previous animal experiments have shown that G-CSF promotes the migration of mesenchymal cells to the site of SCI and inhibits neuronal apoptosis to improve functional recovery.^213^ G-CSF was first studied in a clinical trial on SCI by researchers from the Inha Neural Repair Center, who combined CSF and autologous bone marrow mesenchymal stem cells (MSCs) to treat complete SCI. The preliminary assessment showed that acute and subacute treatment increased the ASIA scores of patients by 30.4%.^246^ In a recent phase I/IIa clinical trial specifically aimed at studying the safety and effectiveness of G-CSF in patients with SCI, satisfactory recovery of neurological function and improvement in ASIA motor scores were observed in patients.^247^ Currently, the clinical results are not sufficient and convincing. In the future, large-sample, multicenter, randomized controlled studies should be conducted to evaluate the clinical translation value of this molecule.

##### Ganglioside

Ganglioside (GM-1), a complex glycolipid that is abundant in mammalian cell membranes, was once proposed as the most promising medicine to replace glucocorticoids because of its ability to promote axonal regeneration and neuronal cell survival.^248^ GM-1 can downregulate caspase-3 expression and upregulate the expression of NGF following SCI, exerting a therapeutic effect by inhibiting the activation of neuronal apoptotic signals and promoting axonal regeneration. Bose et al. reported that GM protects the continuity of axons after SCI, prompting people to focus on this medicine and subsequently triggering several in-depth clinical studies on it.^249^ A phase I clinical trial designed to assess the effectiveness of GM-1 treatment in patients with SCI reported that GM-1 promotes neurological functional recovery and improves the Frankel score, ASIA score, and Functional Independence Measure.^250^ However, a large-scale phase III trial involving more than 750 patients at 28 institutions with the highest level of evidence failed to reach the researcher’s ambitious expectations. The trial showed that GM-1 did not significantly improve the primary outcome measures and only resulted in partial neurological recovery and a trend toward bladder/rectal function recovery.^251^ Therefore, controversy remains regarding whether GM-1 can effectively treat acute SCI. The difference between the outcomes of preclinical experiments and clinical trials has focused us to consider the therapeutic mechanism of GM-1 and explore the reasons underlying the differences in outcomes in clinical trials, such as differences in administration time after injury and dose and indications for this drug.

##### Chondroitinase ABC (ChABC)

ChABC, an inhibitor of CSPGs, eliminates CSPG glycosaminoglycan (GAG) chains.^95^ After SCI, in addition to destruction of the inflammatory microenvironment by local cascades, the development of a scar containing CSPG inhibits regeneration. CSPGs can inhibit the growth of axons and may hinder the formation of new axons in the CSPG-rich microenvironment. ChABC alters this regeneration-inhibiting microenvironment by significantly degrading CSPG, thereby promoting the growth of axons to induce the reestablishment of upper and lower functional connections. Researchers from the Fawcett laboratory first studied the role of this molecule in improving the local inhibitory environment by injecting ChABC into the SCI site.^95^ They proved that ChABC provides a permissive environment for axonal regeneration (ascending sensory projections and descending CST axons) by increasing the expression of regeneration-promoting proteins and inducing the reestablishment synaptic activity above and below the injured site and restoration of locomotor and proprioceptive functions.^215–217^ Some research has also confirmed that the combination of ChABC and cell transplantation can effectively treatment SCI.^252,253^ Clinical translational research on the efficacy of ChABC alone in the treatment of SCI has not yet been carried out. An evaluation of the safety and effectiveness of ChABC treatment in humans is also needed. With the support of the Spinal Research Charity Fund, the “humanized” form of the ChABC enzyme is actively being prepared through genetic modification, which may provide significant and profound value for future clinical translational research.

#### Neuroregeneration-activating medicines

The main goal of SCI therapies is the promotion of axonal extension through the injury site, allowing the upper CNS to control lower body function. However, some injury-dependent regulatory mechanisms, such as cytoskeletal disorganization, activation of inhibitory intrinsic growth signals, and RNA processing, might lead to failure of axonal regeneration.^73^ Neuroregeneration activators can improve the microenvironment, making it conducive to nerve reconnection. On the one hand, neuroregeneration-activating medicines can specifically target the inhibitory components released into the microenvironment after injury, such as myelin basic protein and ECM proteins, which are critical for eliminating inherent negative factors. On the other hand, neuroregeneration-activating medicines can also stimulate remodeling and the migration of glial cells, which allowing remodeling of the imbalanced structural, physical and chemical environment. Many previous studies have revealed that some medicines affect the regenerative ability of injured neurons, promoting their regeneration.

##### Rho antagonists & Rho-associated kinase inhibitors

Some molecules that are produced after SCI activate the Rho pathway, exerting an undesirable effect on axonal regeneration and neurite growth.^254^ Dergham et al., a group from Canada, were the first to explore the role of the Rho signaling pathway in repair of the injury spinal cord and published a groundbreaking report on the beneficial effects of Rho-associated kinase inhibitors in inducing reconstruction of neurological structures.^254^ Animal experiments have shown that cethrin, a recombinant VX-210 protein that inhibits the RHOA pathway, leads to improved behavioral outcomes and enhanced axonal regeneration after SCI.^219^ An initial clinical trial revealed that early decompression surgery and simultaneous intrawhite matter administration of cethrin significantly improves patients’ ASIA scores without causing adverse reactions.^220^ A phase I/IIa clinical study on cethrin reported that this medicine effectively improves motor function in patients with SCI, and a phase IIb/III trial is underway.^221^ Although the results obtained to date are encouraging and exciting, limited motor function recovery has been achieved, possibly because the medicine has only a single target. More recently, Stern et al. reported that the mechanism of Rho-associated kinase inhibitors seems to be far more complicated than we thought. When Rho is knocked out in neurons, axons regenerate. However, ablation of Rho in astrocytes may induce reactivation and astrogliosis. This emphasizes and confirms the value of neuron-specific RhoA ablation for the treatment of neuroregeneration.^255^ Future clinical translational research may assess whether the medicine shows more significant therapeutic potential when administered in combination with other treatments.

##### Nogo-A antibodies

Caroni and Schwab showed that myelin-related molecules restrain axonal growth in 1988.^256^ Proteins released by the lysis of the myelin sheath after SCI inhibit neurite growth and the development of an inhibitory microenvironment after injury, and Nogo-A is an important myelin-derived protein. As one of the most potent myelin-derived regeneration inhibitors, Nogo-A negatively affects the remodeling of the cytoskeleton and the growth of surviving neurons post-SCI. Researchers have designed antibodies that can specifically block glycoproteins, which greatly antagonize the inhibitory microenvironment induced by Nogo-A. Nogo-A antibodies can promote the survival of neural elements to allow axonal sprouting and induce the reorganization of neural networks in SCI models.^257^ Additionally, robust regeneration of the CST was observed in marmoset monkeys administered a Nogo-A antibody in a study by the Schwab group.^258^

A phase I clinical trial on the application of a humanized Nogo-A antibody (ATI355) for the treatment of SCI started in 2006 with funding from Novartis (ClinicalTrials.gov, Identifier: NCT00406016). The clinical trial included 52 patients with SCI and evaluated the acute safety, feasibility, tolerability, and pharmacokinetics of six dose regimens of ATI355 in patients with acute SCI. However, the results have not yet been announced. Subsequently, a placebo-controlled, randomized, double-blind, multicenter, multinational phase II clinical trial (identifier: NCT03935321, ClinicalTrials.gov) designed to assess the safety, tolerability, feasibility, and preliminary efficacy of early (within 4–28 days postinjury) treatment with repeated bolus injections of a Nogo-A antibody (NG-101) in patients with cervical acute SCI was initiated in 2019 and is expected to be completed in 2023. Furthermore, a first-in-man study of the efficacy of intrathecal administration of a Nogo-A antibody showed that this antibody significantly improves motor scores in patients with acute SCI.^223^ Therefore, in-depth evaluation of the molecular mechanisms in this pathway and strategies targeting the critical components in the intracellular pathway will be the value of the future clinical translation of Nogo-A antibodies.

##### Fibroblast growth factor

Fibroblast growth factor (FGF) is a tyrosine kinase receptor ligand that exerts a significant effect on embryonic development, and its role in promoting axonal regeneration and inhibiting the inflammatory response has been studied.^259,260^ The role of FGF in resisting the destruction of the inflammatory microenvironment and inhibiting programmed cell death has been recognized. In addition, this factor can induce structural remodeling after SCI, mainly by recruiting and stimulating the proliferation and migration of glial cells. In addition to increasing the levels of regeneration-promoting factors and matrix components, FGF provides suitable conditions for neurite outgrowth. A preliminary phase I clinical trial showed that FGF may increase ASIA motor scores, and a phase I/II trial, the conclusions of which have not yet been published, has been performed.^224,225^ FGF is a mitogen that induces cell proliferation and stem cell self-renewal. As FGF may promote stem cell proliferation, it can be used in combination with our agents for SCI treatment in future clinical translational studies.

##### Neurotrophins

There are three representative neurotrophins: brain-derived neurotrophic factor (BDNF), nerve growth factor (NGF), and neurotrophin-3 (NT-3). Due to differences in tyrosine kinase expression, the sensitivity of different types of neurons to specific neurotrophins differs. BDNF promotes the regeneration and sprouting of axons in the reticulospinal, rubrospinal, and vestibulospinal tracts.^261^ Although BDNF was found to promote long-distance axonal regeneration and remyelination in preclinical studies, the poor efficacy and unexpected side effects of BDNF limit its future clinical translation.^262^ Persistent improvements in strategies for the delivery of these molecules, such as virus-mediated gene delivery and intraparenchymal protein infusion, are essential for the integration of the molecules into clinical treatment strategies in the future. In 1990, researchers at the Max Planck Institute first isolated NT-3.^263^ NT-3 administration has the most beneficial effect on the CST, and axonal sprouting is not observed in other tracts, such as the cerebrospinal and rubrospinal tracts.^264^ Current NT-3-based clinical trials mainly focus on neuropathic pain and allodynia-related peripheral neuropathies.^264,265^ NT-3 inhibits the degeneration of peripheral sensory axons and improves their functions. Notably, some patients with SCI suffer from hyperesthesia and neuropathic pain. Future studies on NT-3 aimed at improving sensory function may provide hope for future treatment. Rita Levi-Montalcini was the first to isolate NGF from mouse sarcoma cultures in the 1950s.^266^ It is worth noting that NGF can effectively activate the intrinsic regeneration signals in surviving neurons in the microenvironment after injury, such as MAPK/ERK and PI3K/Akt signals. Activation of the downstream cascade further induces the modification and activation of the cytoskeleton at the growth cone, which is a key for promoting regeneration. Preclinical research has proven that NGF is the main contributor to the regeneration and sprouting of α-motor axons and nociceptive axons.^267,268^ However, NGF may be applied in the clinic to limit nociceptive sprouting and concurrent neuropathic pain in the future, but more clinical trials are needed.

#### Other medicines

When it occurs at or above the thoracic level, SCI disrupts the sympathetic innervation of the CNS, causing coronary contraction and impairing cardiac function. Furthermore, patients are prone to neurogenic shock due to vasodilation below the SCI.^269^ Therefore, the maintenance of arterial blood pressure after SCI is essential. Continuous hemodynamic monitoring and maintenance of a mean arterial pressure between 85–90 mmHg is recommended for the first seven days after SCI according to the AANS/CNS.^270^ Intravenous use of vasopressor drugs, such as norepinephrine and dopamine, significantly improves the local spinal cord perfusion pressure and hemodynamic parameters after SCI.^271,272^ In addition, treatment with mannitol can be considered in the early stages of SCI if local edema or elevated pressure is present.^273^ This drug effectively promotes edema resolution through diuresis, thereby improving the patient’s symptoms. Neurotrophic drugs, microcirculatory modifiers, and excitatory glutamatergic receptor blockers can also improve the neurological prognosis of patients with SCI.^274^

#### Future directions

The treatment time window, individual differences, the degree of injury, and other factors must be considered for rational and combined use of drugs for SCI treatment. Advancements in research on and translation of new medicines for SCI treatment are ongoing. However, researchers must overcome many challenges and difficulties. The inconsistency between results from preclinical trials and those from clinical trials and a lack of scientific research funding are obstacles to large-scale clinical trials. In addition, most drugs achieve nerve regeneration and functional recovery through a single mechanism and a single target, limiting their application value for recovery of spinal cord function after injury. Based on this information, studies that explore the unknown and complex pathological mechanisms of SCI combined and screen therapeutic drugs for multitarget, multimodal, multistage interventions may have good clinical application value and may be translated to the clinic in the future. Simultaneously, the combination of biological tissue engineering or cell transplantation strategies with drugs that exert specific curative effects may provide a good framework for treating SCI.

### Cell therapy

**Fig. 6:**
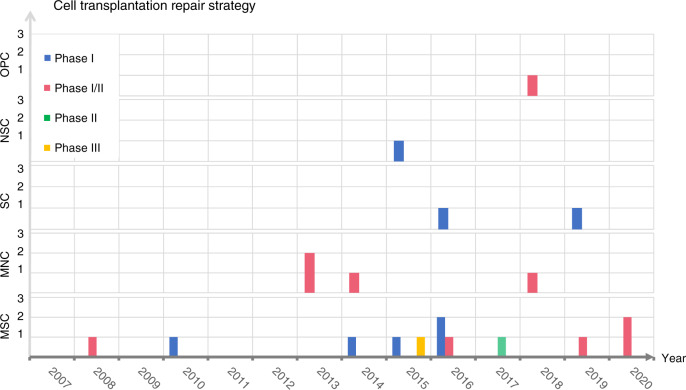
Research progress on cell therapy for spinal cord injury. This figure shows cell therapy clinical trials for SCI that are registered at ClinicalTrials.gov or have been published. This figure shows the timeline of cell therapy research for SCI and the number of clinical trials for different cells

Cell therapy is a promising strategy for SCI treatment. Cell therapy involves various mechanisms, such as nutritional support with multiple molecules that support neuroprotection. These factors may enhance host cell survival, regulate gliosis and inflammation and/or improve vascular regeneration.^275,276^ In general, cell transplantation mainly achieves replacement of spinal cord tissue and promotes axonal growth and myelination and ultimately functional recovery.^277–281^ Clinical trials of various phases on cell transplantation therapy are also in progress. In this review, we focus on clinical trials conducted in recent years. Table 1 lists some representative clinical trials on cell transplantation performed in recent years (Fig. 6, Table 2).

**Table 2:** Cell therapy for spinal cord injury

Cells	Microenvironment regulation	Program	Research object	Effectiveness	Safety/Adverse effects
Autologous mesenchymal stem cell	Secrete nutritional factors, anti-inflammatory, anti-toxic, anti-apoptotic, immune regulation, and promotion of angiogenesis	Oh et al. (Phase III)^282^	16 subjects with traumatic cervical SCI	2 (12.5%) of the 16 patients showed improvement in motor grade of the upper extremities; no changes were noted in the other 14 patients (87.5%) at the 6-month follow-up	No serious adverse reactions;8 patients experienced mild adverse reactions (paresthesia, muscle stiffness, etc.), and these symptoms were relieved within a few months
Autologous human Schwann cell	Secrete nutritional factors, anti-inflammatory, remyelination, regulation of extracellular matrix and inhibition of scar formation	Anderson et al. (Phase I)^283^	6 subjects with subacute thoracic SCI	No change in bladder and bowel control;subjects converted to AIS B had detectable motor evoked potentials in both legs at 6 months and 12 months	No clear indications of adverse events related to nerve harvesting, the cell transplantation process, or the presence of cells in the spinal cord
NSI-566 neural stem cell line	Secrete nutritional factors, anti-inflammatory, inhibition of apoptosis and the formation of glial scars.	Curtis et al. (Phase I)^284^	4 subjects with T2-T12 SCI	3 subjects have improved sensory and motor function;1 subject with no any change, the quality of life scores of all subjects did not change	No immediate or delayed complications; no new spinal cord or soft tissue edema, swelling development, or fluid accumulation after surgery
Human neural stem cell	Secrete nutritional factors, anti-inflammatory, inhibition of apoptosis and the formation of glial scars	Levi et al. (Phase II)^285^	31 subjects with chronic cervical SCI	There is a trend of UEMS and GRASSP exercise gains among the treated participants, but the magnitude is below the required clinical efficacy threshold	No new spinal cord injury or new injury was found
Umbilical cord blood mononuclear cell	Regulation of inflammation, promotion of pericyte migration, reduction of scar formation, and promotion of axon regeneration	Zhu et al. (Phase I–II)^286^	28 subjects with chronic complete SCI	Some patients’ independence in activities of daily living increased, more than half of the patients resumed walking with or no assistance	22 patients had adverse events (neuropathic pain, hyperthyroidism, hypertension, and etc.)
Autologous olfactory ensheathing cells	Remyelination, nutritional support, neuroprotection, promotion of axon regeneration, regulation of extracellular matrix, promotion of angiogenesis, and inhibition of scar formation	Tabakow et al. (Phase I)^287^	6 subjects with chronic thoracic SCI	Patients undergoing surgery have improved spinal cord transmission and lower extremity muscle activity	No evidence of nerve deterioration, neuropathic pain, nerve infection, or tumor formation

#### Neural stem cells

There has been much research on NSCs. NSCs regulate the microenvironment to repair the injured spinal cord through the following mechanisms. 1. NSCs differentiate into neurons to replace dead neurons. A study demonstrated that human NSCs overexpressing BDNF (F3.BDNF) can express the neural marker NeuN after being transplanted intrathecally after thoracic spinal cord contusion.^288^ 2. NSCs express nutrient factors and inhibit cell apoptosis. NSCs expressing acid fibroblast growth factor can inhibit the caspase 12/caspase-3 pathway, the EIF2α–CHOP pathway, and GRP78 protein expression to suppress apoptosis.^289^ Additionally, NSCs can release neurotrophic factors such as GDNF and BDNF.^290^ 3. NSCs inhibit inflammation. Human spinal cord-derived neural stem/progenitor cells (Hsc-NSPCs) can release NT-3 in the injury site and reduce the expression of CD68, IL-1β, and IL-6.^291^ Another study demonstrated that NSC transplantation reduces the expression of proinflammatory cytokines, including IL-1β, IL 6, IL 8, MCP 1, iNOS, and TNF α, which suggests that transplanted NSCs can antagonize M1 macrophages after SCI.^292^ In addition, Melania Cusimano showed that SC-NSC-deficient mice exhibit significant microglial activation and a reduction in oligodendrocyte number.^293^

In 2002, Ogawa et al. showed that transplanted in vitro expanded NSCs can repair the injured spinal cord. They transplanted NSCs into the injured spinal cords of rats on the 9th day after SCI, and the donor-derived neurons extended their processes into the host tissue and formed synapses at five weeks. The treatment group showed better functional recovery than the control group.^294^ Additionally, transplanted NSCs replace lost tissue, suppress inflammation, promote myelin regeneration, proviede neuroprotection and nutritional support, etc.^295,296^. Curtis et al. conducted a phase I clinical study in which human spinal cord-derived NSCs (NSI-566) were transplanted into 4 patients with T2 to T12 injury. After 18–27 months of observation, no serious adverse reactions were found, and improvements in sensation and function were observed in two subjects.^297^ Another phase II clinical study (2 cohorts, 12 patients) published in 2019 showed that human NSC (HUCNS-SC) transplantation is safe in patients with chronic C5–7 injury and quadriplegia leads to functional recovery^285^; however, this study was terminated early. Although NSC transplantation has been shown to be relatively safe and effective in clinical trials,^285,297^ the ethical problems associated with the cell source limit the application of NSCs.^285,297,298^ The use of induced pluripotent stem cells (IPSCs) and cell reprogramming seem to overcome this problem.^299,300^

#### Mesenchymal stem cells

MSCs have a wide range of sources, such as bone marrow (BMSCs), cord blood or the umbilical cord (UCMSCs), adipose tissue (ADMSCs), and the amniotic membrane or amniotic fluid (AFMSCs). Therefore, they are not subject to ethical restrictions.^281,301^ Generally, the main function of MSCs is microenvironmental regulation, as they can antagonize inflammation, protect neurons, promote axonal growth and inhibit scar formation.^301^ 1. MSCs promote axonal growth. BMSCs promote axonal growth by secreting ECM proteins^302^ and trophic factors (VEGF, GDNF, BDNF, NGF, LIF, IGF-1, TGF-β1, etc.).^303–305^ 2. MSCs regulate inflammation. BMSCs promote the transformation of M1 macrophages into M2 macrophages,^306^ and AFMSCs have anti-inflammatory effects.^307^ 3. MSCs suppress apoptosis. UC-MSCs are thought to downregulate the expression of proapoptotic factors (BCL10 and BCL10), mitochondrial apoptosis-related genes (Bad, Bid, Bid3, Bik, and Bak1) and also downregulate the expression of caspase genes, as well as genes associated with the NF-κB apoptosis pathway (TNF-A, TNFR1, TNFR2, Fas, LTA, and CD40).^308^ 4. In addition, Daniele Bottai’s research showed that AFMSCs can promote angiogenesis and increase the mRNA expression levels of vascular endothelial growth factor and hypoxia inducible factor-1α after transplantation.^309^

In recent years, some advancements have been made in clinical research on MSCs. Many experiments have proven the safety and effectiveness of MSCs in the repair of the injured spinal cord.^246,310–312^ In an open-label, nonrandomized phase I/II clinical trial including 35 patients with complete SCI, BMSCs were injected into the SCI site at different time points. Except for exacerbation of injury in a small region at the cell transplantation site, no bleeding, infection, or new lesion formation was observed. A total of 30.4% of the patients in the acute and subacute groups had higher AIS scores than the patients in the chronic group. In contrast, the chronic group showed no significant improvement in AIS scores.^246^ A phase III clinical trial performed by Oh et al. demonstrated the safety and limited effectiveness of autologous MSCs. In this study, 16 patients who received autologous bone marrow MSC injection did not experience any adverse reactions related to stem cell injection, but only two patients showed functional improvement.^310^ Regarding UC-MSCs, patient registration was recently initiated for an ongoing multicenter randomized, sham-controlled phase II trial (NCT03521336) evaluating the efficacy of intrathecal transplantation of UC-MSCs. The study is expected to be completed in 2022.^281^ In clinical studies on ADMSCs, most patients reported a slight improvement after intrathecal transplantation, but longitudinal clinical trials studying specific motor responses are lacking.^277^ Moreover, clinical trials of the efficacy of AFMSCs in treating SCI are lacking.^281^

Although some advancement have been made in the application of MSC transplantation for the treatment of SCI, some clinical challenges persist.^281^ Although the safety of MSCs has been proven, their efficacy is relatively limited.^246,310,312,313^ Additionally, financial constraints and ethical and logistical constraints are responsible for the lack of large-scale phase III trials.^281^ Specific and optimal treatment strategies involving MSCs must be further explored.^281^

#### Schwann cells

SCs transplantation has been used to treat SCI for a long time. In 1980, I.D. Duncan et al. transplanted SCs cultured in vitro into the demyelinated area of the spinal cord. At 2–18 weeks after transplantation, remyelination by SCs was observed.^314^ SCs regulate the microenvironment to repair the injured spinal cord through the following mechanisms. 1. SCs promote axonal regeneration and myelination.^315,316^ 2. SCs express growth factors. SCs can promote the recovery of spinal cord function through a variety of molecular mechanisms, such as the production of a variety of growth factors (including NGF, BDNF, and CNTF), cell adhesion molecules (N-CAM, N-cadherin, and integrins), and ECM proteins (collagen and laminin).^278,317,318^ 3. SCs inhibit the activation of microglia and astrocytes. A study confirmed that the transplantation of SCs reduces the number of Iba-1^+^ and GFAP^+^ cells at the site of SCI and the expression of mitochondrial division markers (FIS1) and increases the expression of mitochondrial fusion markers (mfn1 and Mfn2) in the injured spinal cord.^319^

In a phase I clinical trial, Anderson et al. transplanted autologous SCs into patients with subacute SCI. The results revealed no surgical, medical, or neurological complications one year after transplantation, no adverse events or serious adverse events related to cell therapy, and no evidence of additional SCI, mass damage, or cavity formation. These results indicate that SC transplantation is a safe treatment for SCI.^283^ Our group also conducted a clinical study. Six patients with SCI who received autologous SC transplantation were followed for more than five years. All patients showed some improvement in autonomic, motor, and sensory functions. Therefore, autologous activated SC transplantation may be a feasible, safe, and effective method to promote nerve repair in patients with SCI.^320^ Similar to MSCs, SCs have been proven to be safe, but their effectiveness is relatively limited.^283,320,321^ Moreover, combination therapy is more effective than SC transplantation alone, which may be a future direction.^322^

#### Induced pluripotent stem cells

IPSCs are similar to embryonic stem cells (ESCs) because they produce cells from all three germ layers. Therefore, iPSCs can be cultured and induced to enrich ectodermal neural cells. In addition, no ethical problems are associated with the application of iPSCs, which can be obtained from human somatic cells.^323^ Studies have confirmed that iPSCs improve the local microenvironment after SCI. 1. iPSCs reduce inflammation and inhibit scar formation. After hiPSC transplantation, the number of local astrocytes, microglia and macrophages is reduced; GDNF, TNF-α, VEGF, IL-10, and MIP-1-α are expressed, and the deposition of chondroitin sulfate around the injury site is reduced; these findings suggest that iPSCs can promote the recovery of tissue and improve the microenvironment of the injured spinal.^324^ 2. iPSCs can differentiate into neurons and replace cells. During the repair of the injured spinal cord, iPSCs mainly promote functional recovery by differentiating into neural precursor cells, neural crest cells, neurons, oligodendrocytes, astrocytes and even mesenchymal stromal cells to replace lost cells and/or regulate the lesion microenvironment.^325^

Kobayashi et al. conducted the first study proving the effectiveness and safety of iPSCs in SCI treatment. They used human IPSC-derived neural stem/progenitor cells (HIPSCs NS/PC) to treat a adult marmosets with C5 SCI. Compared with the control treatment, HIPSC NS/PC transplantation promoted axonal preservation, regeneration, and angiogenesis. Additionally, HIPSC NS/PC transplantation prevented demyelination and promoted functional recovery after SCI.^299^ IPSCs differentiate into neurons and form synapses with host neurons. IPSCs differentiate into oligodendrocytes to promote myelin sheath formation and secrete neurotrophic factors to promote functional recovery.^326^ Many studies have documented the safety and efficacy of IPSCs. Kawabata et al. reported that human IPSC-derived oligodendrocyte precursor cells rich in NSCs/progenitor cells promote the remyelination of demyelinated axons and functional recovery after SCI.^327^ Narihito Nagoshi systematically described the current status of the application of iPSCs for the treatment of SCI in a review and indicated that his team is preparing to conduct the first clinical trial on iPSCs as a treatment for subacute SCI and plans to start this study soon.^326^ On the other hand, transplanted IPSCs have the potential to induce genetic and epigenetic abnormalities, tumorigenicity, and immunogenicity, indicating that cell therapy based on IPSCs may pose severe safety-related challenges.^323^ Therefore, clinical research on IPSC transplantation for the treatment of SCI is limited. In the future, more work is needed to avoid these shortcomings.

#### Olfactory ensheathing cells

Olfactory ensheathing cell (OEC) transplantation is a promising treatment for SCI.^328^ As shown in a study by Li et al., OECs promote regeneration and functional recovery of the CST in adult rats with upper cervical SCI.^329^ Repair of the injury spinal cord by OECs involves the following mechanisms: 1. Myelination. Both in vivo and in vitro studies have shown that olfactory ensheathing cells produce myelin components and secrete molecules related to myelination, such as P0, allowing them to form myelin of the appropriate size around demyelinated axons.^330^ 2. Neurotrophin expression. OB-OECs synthesize neurotrophins, such as NGF, BDNF, GDNF and neuregulin,^331^ while OM-OECs secrete a variety of cell adhesion and matrix molecules, such as laminin, fibronectin, and NCAM; both OB-OECs and OM-OECs promote the growth of axons.^332^ 3. Promotion of vascularization. Transplanted olfactory ensheathing cells increase the production of new blood vessels and upregulate COX-2 and VEGF expression in astrocytes.^333^ In recent years, Tabakow et al. evaluated the safety and efficacy of OEC transplantation in a phase I clinical study. Six patients with chronic paraplegia were included in the study, and three of them received OEC transplantation. No adverse consequences related to OEC transplantation were observed 1 year after the operation. No evidence of neurological deterioration, neuropathic pain, nerve infection, or tumor formation was recorded. Patients receiving transplantation showed improved muscle activity in the lower extremities. This study indicates the safety and potential effectiveness of OEC transplantation.^287^ In another phase I clinical trial, Lin Chen and colleagues transplanted OECs or SCs alone into patients with chronic complete cervical SCI. The combination therapy was more beneficial than the control treatment.^334^ A phase I/II clinical study by Lima et al. confirmed that OEC transplantation combined with postoperative rehabilitation is beneficial for patients with chronic SCI. Of the 20 patients who received transplantation, 11 patients showed improved AIS scores.^335^ However, the effectiveness of OEC transplantation must be confirmed before this strategy can be translated to the clinic. Although many experiments have reported the effectiveness of OECs in treating SCI, other experiments have produced unsatisfactory results. Some researchers have reported that only a few patients experience a slight improvement in motor function, and some patients show no improvement at all.^336,337^ Additionally, there are obvious problems related to the invasive nature of the method used to obtain OECs from the patients’ olfactory bulbs. Even if a minimally invasive approach is used, these problems must be considered.^338^

#### Embryonic stem cells

Human embryonic stem cells (hESCs) have excellent differentiation potential and can produce neurons or glial cells for transplantation.^339^ In 1999, McDonald et al. showed that ESCs transplanted into the injured spinal cord of rats differentiated into astrocytes, microglia, and neurons. Compared with the control group, rats treated with ESCs showed a significant improvement in motor behavior.^340^ The mechanism by which ESCs repair the injured spinal cord mainly involves differentiation into neurons and glial cells to replace the lost spinal cord cells and the production of factors that limit injury and promote regeneration.^278^ In 2016, a study involving 226 patients with SCI showed that intramuscular injection of human ESCs was safe and improved the AISA score.^341^ In a recent study, Geeta Shroff and Rakesh Gupta administered human ESCs intramuscularly to five paraplegic patients. After treatment, the sitting posture balance, sense of control of defecation and urination, and limb motor function (lower limbs and upper limbs) of all patients were significantly improved. The differences in these measured after treatment compared with before treatment were statistically significant, and no adverse events were reported.^342^ Although ESCs are the perfect candidates for stem cell therapy, there are some ethical issues related to the use of hESCs because human embryos are needed to obtain these cells. In 2010, Geron proposed a trial involving the transplantation of oligodendrocytes derived from ESCs into the injured spinal cords of patients, but this study was terminated for ethical and financial reasons.^343^ In addition, tumorigenicity is another critical problem that must not be ignored.^344^

#### Macrophages

In 1998, Rapalion et al. transplanted macrophages into rats with thoracic spinal cord transection. Compared with the control group, the transplantation group showed better improvement of motor function and electrophysiological parameters. Histology showed that the nerve fibers crossed the transected spinal cord.^345^ M1 macrophages are predominant in the early stage of CNS inflammation. In contrast, M2 macrophages play a dominant role in the resolution of CNS inflammation, including after SCI, and alternatively activated M2 macrophages contribute to alleviation of SCI.^346^ Additionally, a study showed that M2 macrophages can secrete PDGFB and act on PDGFR β, promoting the migration of peripheral cells after SCI to reduce the formation of scar tissue and promote axonal regeneration.^347^ Clinical studies have also proven the feasibility of macrophage transplantation for the treatment of SCI. In a phase I clinical study on macrophages, three of eight patients who received autologous macrophage transplantation (ASIA grade A) experienced clinically significant recovery of neuromotor and sensory function (ASIA grade C). Although two patients experienced pulmonary embolism and one experienced osteomyelitis during the study period, these events were not considered to be related to macrophage transplantation.^348^ However, M2 macrophages are present in other conditions, such as during tumor progression and intracellular pathogen infection. Although M2 macrophages have a clear role in CNS inflammation, the exact role of these molecules remains to be determined. Further studies are needed to elucidate how M2 macrophages are activated in the context of CNS inflammation.^346^

#### Oligodendrocyte progenitor cells

OPC-mediated repair of the injured spinal cord involves the following mechanisms: 1. Myelination. Nazari et al. showed that mir-219-overexpressing OPCs (mir-219OPCs) can differentiate into mature oligodendrocytes expressing MBP in vivo and promote the recovery of function after being transplanted into the lesion.^349^ 2. Immune response regulation. OPCs can regulate the adaptive immune response by acting as antigen-presenting cells following chemical- and autoimmune-mediated demyelination. In addition, OPCs can produce cytokines (IL-1β, CCL2, MMP9) to regulate the innate immune response after tissue injury, and these cytokines play an important role in the immune regulation of the microenvironment after SCI.^350^ Keirstead et al. reported the feasibility of OPC transplantation for SCI treatment in 2005. They transplanted OPCs derived from hESCs into rats 7 days and 10 months after SCI. In both cases, the OPCs survived and differentiated into oligodendrocytes. However, rats receiving OPC transplantation showed more myelination and better recovery of motor function. This result proves the therapeutic potential of OPCs in early SCI.^351^ After SCI, OPCs differentiate into astrocytes, oligodendrocytes, and SCs, which provide nutritional support, promote the formation of the myelin sheath, and ultimately improve SCI recovery.^350,352,353^ Recently, Manley et al. conducted a preclinical experiment in a rat model of cervical SCI, providing a basis for the application of OPC transplantation strategies for cervical SCI treatment. The researchers injected hESC-derived OPCs into the cervical spinal cords of rats one week after contusion injury. The rats showed significant improvements in motor function, less cavity formation, and more myelinated axons. In terms of safety, the injection of OPCs did not cause any adverse reactions, toxicity, hyperalgesia, or tumors. These results support the initiation of a phase I/IIa clinical trial in the United States for patients with sensorimotor dysfunction after complete cervical SCI.^354^

#### Current stage and future directions

1. Safety. Although some adverse reactions occur in response to cell transplantation, such as paresthesia, muscle stiffness, neuropathic pain, hyperthyroidism, and hypertension, these adverse reactions either resolve naturally or can be treated with drugs. Some of the adverse reactions may not be related to cell transplantation. Therefore, in general, treatment of SCI via cell transplantation is relatively safe.^282–287^
2. Effectiveness. In all clinical trials, most subjects tended to exhibit sensory and motor function recovery, and only a few patients presented no changes. Although sensory and motor function recovery did not meet the corresponding clinical standards in a considerable number of patients, the potential effectiveness of cell transplantation is undoubtable.^282–287^
3. Large-scale trials. Current clinical trials evaluating the efficacy of stem cell transplantation for the treatment of traumatic SCI are progressing slowly, and large-scale phase III trials on the therapeutic effect of stem cell transplantation are lacking. Many factors may contribute to this lack of evidence, including but not limited to ethical challenges related to the use of ESCs and patient acceptance.^277^
4. The best therapeutic strategy. Factors related to the therapeutic effect of cell transplantation must be more accurately identified and studied. As mentioned above, although some researchers have examined the optimal time point for transplantation, dose of transplanted cells, and method of transplantation,^277,286^ related experiments are still lacking.
5. Combination strategies. Further research on the combination of cell therapy and other treatment methods is needed. Previous studies have summarized the efficacy of some combined treatment methods, but significant differences in the effects of these combined treatments have been reported.^277–279,355^ The best combination approach should be identified in the future.
6. New cell candidates. The effectiveness of transplantation of other cell types in the treatment of SCI should also be evaluated. For example, interneuron- and multilineage-differentiating stress-enduring (MUSE) cells have proven to be safe and effective in treating SCI in rats.^356–358^

### Exosomes

The reparative effects of exosome, as noncellular agents, have been studied extensively in recent years. Exosomes, which are functional extracellular vesicles, are secreted by many different cells and play a vital role in communication among cells. The diameter of exosomes is approximately 30–1150 µm, and exosomes express unique markers, such as CD81, CD63, CD9, HSP70, and ALG-2-interacting protein X. The contents of exosomes include proteins, nucleic acids, and lipids.

#### Application of exosomes derived from different cells for repair of the injured spinal cord

Exosomes derived from MSCs are the most studied exosomes regarding repair of the injured spinal cord. Exosomes derived from MSCs can be easily obtained in considerable quantities, and there are no associated ethical restrictions. Huang et al. applied exosomes derived from BMSCs to repair the injured spinal cord and found that the exosomes attenuated apoptosis and inflammation after SCI and promoted functional recovery.^359^ Additionally, another study reported that exosomes derived from BMSCs suppress A1 astrocyte and complement activation.^360^ Exosomes can also be obtained from pericytes. Yuan et al. reported that exosomes derived from pericytes protect the blood–spinal cord barrier and that this effect is associated with the PTEN/AKT pathway. Furthermore, a study showed that exosomes from human placenta-derived MSCs promote functional recovery after SCI.^361^ Similarly, some vesicular exosomes secreted by oligodendrocytes can also have a certain impact on SCI. The contents of these exosomes can affect neuronal gene expression, strengthen neuronal resistance to oxidative stress, and regulate neuronal differentiation through miRNA combined with a microtubule-stabilizing protein (Doublecortin).^362^ Intriguingly, exosomes produced by neurons can be used to repair the injured spinal cord. Exosomes derived from neurons can suppress M1 microglial and A1 astrocyte polarization and promote functional recovery.^363^

#### Role of exosome carriers in repair of the injured spinal cord

Recently, researchers have focused on exosome carriers. In some studies, functional exosomes containing large amounts of special nucleic acids, such as nonlong coding RNAs^364^ and miRNAs,^365^ or loaded with PTEN siRNA^366^ have been generated to suppress apoptosis and inflammation and promote angiogenesis and regeneration. These modifications enhance the functions of exosomes. Furthermore, researchers have aimed to improve the reparative effects of these exosomes by combining exosomes with tissue engineering materials. In one study, Exo-pGel (H-MSC-derived exosomes combined with a peptide-modified adhesive hydrogel) was created and transplanted into the spinal cord. The authors found that exosomes exhibited sustained release and improved the microenvironment.^367^ These modifications increased the local concentration of exosomes and amplified their reparative effects.

#### Future direction

However, no clinical trials using exosomes to treat SCI have been reported. Several issues should be solved before the initiation of a clinical trial. 1. The components of exosomes need to be standardized for clinical application. Currently, MSCs seem to be the best source of exosomes. However, the proteins, nucleic acids, and lipids in these exosomes should be identified, and their levels should be quantified. Our group identified the essential proteins in exosomes derived from SCs through proteomics.^368^ 2. Most researchers have adopted systematic and noninvasive methods for the administration of exosomes. However, only a small amount of exosomes reach the epicenter. Thus, targeted exosomes should be explored.

### Tissue engineering

Tissue engineering strategies combined with cell biology, materials science, and molecular biology methods have been used to repair the injured spinal cord. Generally, combined repair strategy involves various cells, factors or drugs and materials. There are several main mechanisms by which tissue engineering strategies can be used to repair the injured spinal cord. These strategies can be used to regulate the imbalanced microenvironment. Cells, exosomes, or drugs loaded on tissue engineering materials are released into the tissue, and cells further secrete neurotrophic factors or other biologically active small molecules. These methods can also be used to promote intrinsic regeneration ability. Materials or biologically active small molecules interact on neurons or endo-NSCs and promote regeneration, activation or differentiation. Finally, tissue engineering strategies can be used to bridge gaps in injured tissue. The materials can fill the cavity formed during the subacute and chronic phases after SCI and form structural connections.

#### Establishment of a regenerative microenvironment

After SCI, an imbalanced microenvironment is detrimental to nerve regeneration, as mentioned above. Thus, rebalancing the SCI microenvironment is an effective method to repairing the injured spinal cord. Tissue engineering strategies involve three basic components: the material, cells and factors. First, engineering materials can act as a bridge to connect the rostral and caudal sides of the lesion after SCI and provide a pathway through which axons can pass through or enter the scaffolds. Thus, porous channels^369,370^ and fibrous scaffolds^371,372^ have been used to repair the injured spinal cord. Second, tissue engineering materials can rebalance the microenvironment, such as by suppressing glial scar formation and the inflammatory response and promoting remyelination. The material can interfere with cells and affect their survival and differentiation. Moreover, the surface morphology of the material scaffold can induce the biological function of cells to a certain degree. Another characteristic of tissue engineering materials is their biocompatibility, and emerging nanoparticle decellularized tissue matrices have been studied extensively. Decellularized materials, which is are natural materials, have been applied to repair the injured spinal cord. According to Xu et al., decellularized tissue matrices derived from the spinal cord promote functional recovery after SCI. The researchers also identified the key proteins that regulate the behavior of neural stem/progenitor cells.^373^ Additionally, transplanted cells and induction factors are important. Materials loaded with different cell types (NSCs, MSCs, SCs, etc.) have been used to treat SCI. These materials might provide a better environment for the survival of the loaded cells, which can secrete factors such as BDNF and NT-3. Lu et al. used NSCs to treat SCI and found that the NSCs alone did not fill the transection site. Thus, they used fibrin matrices containing a cocktail of growth factors loaded with NSCs. The axons of neurons in the graft extended into the host tissue, and the host axons also extended into the graft. Based on these results, fibrin matrices loaded with NSCs combined with a “cocktail” might be able to modify the imbalanced microenvironment to promote regeneration.^374^ Our team also found that NSCs loaded in polycaprolactone electrospun fiber scaffolds upregulate the expression of NGF and glial cell-derived neurotrophic factor and promote functional recovery in SCI rats.^371^

Advancements in three-dimensional (3D) bioprinting have improved the accuracy of tissue engineering strategies in repairing the injured spinal cord.^375^ 3D bioprinting can be used to easily create bioinks (containing cells, factors, and materials) in different dimensions and shapes. Considering the proliferation and differentiation capabilities of NSCs, these cells have been used as seed cells for the verification of 3D-printed biomaterials in many studies. Furthermore, 3D-printed biomaterials can improve cell viability and are better able to induce the differentiation of NSCs into neurons and the formation of neural networks.^376,377^ Koffler et al. used a microscale continuous projection printing method to make a scaffold with microchannels consistent with the structure of the spinal cord. The results demonstrated that the scaffold could suppress the reactivity of astrocytes. Furthermore, they loaded NSCs into this scaffold and used this platform to repair the injured spinal cord; the spared axons extended into the grafts in the channels and promoted functional recovery.^378^ This study indicates that the precise structure of the scaffold may allow remodeling of the injured spinal cord. Furthermore, a bioengineered spinal cord was generated using IPSC-derived spinal neuronal progenitor cells (sNPCs) and OPCs laid out in a certain manner. The results demonstrated that sNPCs could survive in the channel of the scaffold and differentiate into mature neurons. This method allows the design of different scaffolds.^379^

#### Promotion of endogenous regeneration ability

Engineered scaffolds can also promote endo-NSC activation to achieve spinal cord repair. The safety and effectiveness of collagen scaffolds in repair of the injured spinal cord have been studied for many years. A group utilized linearly ordered collagen scaffolds to repair the injured spinal cord and modified the scaffolds with different factors or drugs, such as N-cadherin and PTX-encapsulated liposomes, to induce the migration of endo-NSCs and promote axonal regeneration. These special scaffolds promote NSC differentiation into neurons and functional recovery.^380,381^ Furthermore, the authors loaded human UC MSCs into collagen scaffolds to treat patients with complete SCI, and the ASIA score changed from A to C after treatment,^382^ indicating that application of these scaffolds is a promising strategy. However, more patients and proper controls are needed to investigate the effectiveness of collagen scaffolds. Another group used NT-3-loaded chitosan to repair the injured spinal cord.^370^ Endo-NSCs were activated and differentiated into neurons. The researchers further analyzed the underlying mechanisms by performing transcriptome analyses and found that the scaffolds regulated neurogenesis, angiogenesis, and inflammation.^383^ Moreover, experimental results obtained from monkeys also verified these results, showing that the regenerating axons could extend through the 1-cm-long lesion.^369^

Although combination tissue engineering strategies show promise in repair of the injured spinal cord, clinical progress has been slow. In 2016, the Neuro-Spinal Scaffold was transplanted into the spincal cord cavity (T12) of a 25-year-old patient with traumatic SCI, and after 6 months of follow-up, no complications were noted.^384^ The Neuro-Spinal Scaffold contains FDA-approved poly(lactic-coglycolic acid) and poly(l-lysine). Another report from the INSPIRE study evaluated the safety of the Neuro-Spinal Scaffold and its ability to promote neurological recovery in patients with incomplete SCI. Kim et al. did not observe adverse events associated with the Neuro-Spinal Scaffold. However, among the 19 patients, 8 patients showed an increase in the level of neurological level of 1–4.^385^ Although several clinical trials have been registered at clinicaltrials.gov, some of them have not been completed.

#### Future directions

Currently, the use of scaffolds loaded with cells and factors may be the most promising strategy for repair of the injured spinal cord. However, much more work is needed to apply this strategy in the clinic. First, many studies are needed to identify suitable scaffolds for repair of the injured spinal cord according to the degree and type of SCI. Furthermore, the technology used to evaluate the injured spinal cord (the type of SCI, the size of the cavity, sparing of axons, etc.) must be improved. With the development of 3D printing, more precise scaffolds can be created to improve anatomical structure. Moreover, better designed large-scale clinical trials should be conducted, especially those that control the study and the outcomes.

### Reprogramming

#### Cell reprogramming after SCI

Cell reprogramming technologies have been used to acquire new neurons after SCI. Astrocytes, fibroblasts,^386^ and NG2 glia have been reprogrammed into neurons in many studies. After SCI, astrocytes and NG2 glial cells are activated and generate new astrocytes. Thus, reprogramming of astrocytes into neurons is a potential strategy for repairing the injured spinal cords. Ascl1 or Neurog2 reprogram astrocytes into neurons.^387^ However, some studies have reported that SOX2 is sufficient to reprogram astrocytes^388^ and NG2 glial cells^389^ into neurons. Su et al. injected a lentivirus (hGFAP promoter) expressing GFP-T2A-SOX2 into the lesion site after severe SCI. DCX^+^, GFP^+^, and TUBB3-expressing cells were detected, indicating that SOX2 induced neurogenesis. Furthermore, new neural cells were shown to be derived from astrocytes in map-Cre;Rosa-tdT mice.^388^ However, only 3%–6% of GFP^+^ cells in the injection site were reprogrammed. SOX2 has the ability to reprogram cells into progenitor cells. Another method has been developed to induce fibroblasts to differentiate into spinal motor neurons. Son et al. forced the expression of seven factors (Ascl1, Brn2, Myt1l, Lhx3, Hb9, Isl1, and Ngn2) in fibroblasts and induced fibroblasts to differentiate into cells with a neuronal morphology. These cells had a motor neuron gene expression profile.^390^ Researchers have further explored the efficiency with which astrocytes are reprogrammed into neurons. Zarei-Kheirabadi et al. found that Zfp521 was more efficient at inducing astrocytes to differentiate into neurons than SOX2 in vitro.^391^ However, the results obtained from in vivo experiments were the opposite. Other cells can also be reprogrammed into neurons. A study reported that NeuroD1 can directly induce the conversion of microglia into neurons.^392^ Matsuda et al. used lentiviruses or shRNA to control the expression of eight translation factors (Ascl1, Brn2, Myt1l, Olig2, Zic1, Sox2, Neurog2, and ND1) in microglia and found that many microglial cells expressed neuron-specific markers. Furthermore, these reprogrammed cells could form excitatory synapses with primary cultured neurons. Moreover, this technology could also be used to reprogram pericytes into neurons.^393^ Karow et al. used a retrovirus to regulate the expression of the transcription factors Sox2 and Mash1 to acquire neuronal cells from pericytes. At present, introducing foreign genes into cells using viral vectors is the main method to induce reprogramming. In general, lentiviruses and retroviruses can be used to directly and effectively integrate genes into the genome of host cells and achieve appropriate expression levels of TFs. However, the shortcoming of this method is the induction of neoplastic gene mutations. Moreover, the introduction of ectopic genes limits the therapeutic application of induced neurons. In addition to this method, combinations of some small molecules can also be used to successfully induce cell reprogramming. Hu et al. used a chemical cocktail including 0VPA, CHIR99021, Repsox, forskolin, SP600125, GO6983, and Y-27632 to successfully reprogram fibroblasts into GABAergic, cholinergic, and dopaminergic neurons.^394^ Some researchers also believe that CRISP/cas9 gene editing can be used to upregulate the expression of or silence relevant transcription factors to achieve accurate reprogramming.^395^

#### Future directions

Currently, there have been no clinical trials on the use of reprogramming technologies to treat SCI. However, some scholars have systematically proven that overexpression of NeuroD1 or knockdown of ptbp1 cannot transdifferentiate astrocytes into neurons in vivo by using a pedigree tracking method. This shows that this method is not feasible in the clinic, and there are many problems to be addressed.^396^ 1. The safety of reprogramming and other gene therapies must be evaluated, which is the main obstacle for their application for SCI treatment. Although astrocytes and fibroblasts can be reprogrammed into neurons, their effects on the microenvironment have not been well studied. The elimination of astrocytes does not promote regeneration after SCI.^100^ 2. Neurons derived from astrocytes and other cells should be further characterized, and their functions should be evaluated.

### Rehabilitation

The role of rehabilitation in SCI treatment has become increasingly prominent in recent years. Appropriate rehabilitation training helps ameliorate cardiorespiratory dysfunction and prevent muscle atrophy after SCI, suggesting its importance in improving remaining functions and restoring lost functions.

#### Rehabilitation strategy for regulation of the microenvironment in the acute and subacute phases

In the acute and subacute phases after SCI, the inflammatory microenvironment becomes dominant. Inflammation induces cell death and activates astrocytes, microglia, and fibroblasts. Therefore, at present, the main goal of acute rehabilitation strategies is reducing the inflammatory response. Hyperbaric oxygen (HBO) therapy can diminish oxidative stress, reduce inflammation, and prevent spinal cord edema, thereby promoting neurological recovery.^397,398^ One study explored the mechanism underlying the effectiveness of HBO in repairing the injured spinal cord. An in vitro study showed that HBO can induce the expression of heat shock protein (HSP) 32 and that ROS/p38 MAPK/Nrf2 play an important role in this regulatory process.^399^ Some studies have proven that HBO therapy is an effective treatment for patients with acute SCI and that the earlier the treatment is administered, the better the effect.^400,401^ Therefore, emergency treatment for SCI should be involve HBO therapy in the hospital.

#### Rehabilitation strategy for regulation of the microenvironment in the chronic phase

In the chronic phase of SCI, the inflammatory response tends to be stable, and neurotrophy and regeneration become key to repairing the injured spinal cord. In particular, neurotrophic factors regulate nerve regeneration and nerve plasticity.

Notably, early locomotor training exerts a positive effect on axonal regeneration by inducing the release of some beneficial cytokines and altering cellular signaling (such as IGF-1 signaling).^402,403^ Representative strategies including Kunming locomotor training and the Kunming locomotor scale (KLS), which were established by Zhu et al. of Tongren Hospital in Kunming, China. Clinical research on the effectiveness of umbilical cord blood-derived mononuclear cell (UCB-MNC) transplantation for the treatment of SCI indicates that UCB-MNC transplantation coupled with Kunming locomotor training results in significant improvements in WISCI and SCIM scores.^286^

Additionally, activity-based therapies,^404^ which are emerging rehabilitation methods for patients with SCI, are currently being accepted and recognized by an increasing number of scientists and clinicians. The concept emphasizes that nerve plasticity is a prerequisite for rehabilitation. Simultaneously, neuromuscular activation and repetitive locomotor training results in satisfactory rehabilitation after SCI. Functional electrical stimulation (FES) is a promising strategy for restoring the ability to walk in paralyzed patients with SCI. FES allows the injured spinal cord to be bypassed and the brain and muscle to be connected, the spared region of the nervous system plays an important role in this process. FES facilitates spontaneous movement, muscle strength, and function in patients with SCI during the recovery period and improves neurological prognosis.^405^ FES also plays a significant role in ameliorating the imbalance of the microenvironment following SCI by activating the inherent regeneration program of neurons and remodeling unstable structures. FES is a neuromodulatory regenerative strategy that activates specific developmentally regulated signaling pathways associated with axonal regrowth. FES promotes mTOR and Jak/Stat signaling in the CST system, which can effectively reactivate axonal sprouting and synapse formation.^64^ This causes damaged axons to return to their original active growth state. In addition, this repair strategy promotes the restabilization of damaged structures in the lesion area. Secondary injury after SCI is accompanied by the activation of calpain and caspase-3, which initiates apoptosis.^406^ Application of electrical stimulation reverses this process and increases the survival of neurons. The effect of epidural spinal cord stimulation in protecting myelin after injury and promoting the differentiation and survival of oligodendrocytes has also recently received attention. These findings also provide evidence for the ability of FES to improve the microenvironment after SCI.^407^

Preclinical and clinical research has shown that stimulating local spinal circuits induces constant functional improvements.^408^ This method is a prospective approach to enhance axonal regeneration and reinforce remaining neural connections within the spinal cord.^409^ FES can accurately stimulate specific nerve roots temporally and spatially. Temporally and spatially specific stimulation combined with 1–3 months of locomotor training may prevent the loss of proprioceptive information, significantly restoring muscle strength and coordination of the lower extremities and improving gait consistency.^410^ Courtine et al. performed numerous studies assessing the effectiveness of brain-computer interfaces and FES for the treatment of SCI. They used an implanted pulse generator with real-time triggering capabilities to re-establish adaptive control of paralyzed muscles after SCI in animals and primates.^411,412^ In 2018, they published their results from human trials. The study included three patients. FES promoted neurological recovery and improved activities of daily living.^413^ Furthermore, Greiner et al. explored the mechanisms by which FES induces upper limb motor neuron recruitment via cervical circuits and found special fiber populations recruited by FES. These results accelerated the application of FES in the treatment of cervical SCI.^414^ However, in the future, rehabilitation aids should be comprehensively selected according to the level and degree of SCI, the patient’s general condition, and the goal of rehabilitation to achieve the optimal treatment effect.

#### Future directions

Rehabilitation therapies need to be applied throughout the period after SCI. At present, rehabilitation treatment strategies for SCI are advancing rapidly. However, there are still some problems to be addressed. 1. The repair mechanism of current rehabilitation strategies needs to be further studied. In-depth analysis of the microenvironmental regulation mechanism of rehabilitation strategies will aid in the development of a complete and effective rehabilitation program. 2. Clinical research on rehabilitation strategies is lacking and is still in its infancy, and such research requires large samples for verification. Moreover, different rehabilitation programs are required for spinal cord injuries of different degrees. For example, the effect of FES on incomplete SCI needs to be evaluated.

Undeniably, great advancements in preclinical and clinical research on SCI has been made, especially in the field of basic research. The composition of the spinal cord is very complex. Due to advances in basic research technologies, an increasing number of cell types and the interactions between cells in spinal cord tissue have been discovered, and the regenerative capacity of the spinal cord has also been explored in depth. However, the pathological mechanism of SCI remains to be clarified and described more accurately. The imbalanced microenvironment dynamically changes after SCI, and thus, use of a single target or single strategy is not sufficient to treat the disease. Recently, multitarget and multitemporal treatment strategies for SCI have been studied. Although some treatment strategies that have achieved good results have been clinically translated in individuals with SCI, there are still many challenges related to the development of clinical trials and multicenter clinical trials, such as ethical issues, trial design issues, and sample size issues. The results achieved at this stage (via gene regulation, brain-computer interface, epidural stimulation, etc.) have provided great hope.
